# A Review of Advances in Bioanalytical Methods for the Detection and Quantification of Olanzapine and Its Metabolites in Complex Biological Matrices

**DOI:** 10.3390/ph17030403

**Published:** 2024-03-21

**Authors:** Anna Czyż, Alicja Zakrzewska-Sito, Julita Kuczyńska

**Affiliations:** 1 Chair of Analytical Chemistry, Faculty of Chemistry, Warsaw University of Technology, 00-664 Warsaw, Poland; anna.czyz.dokt@pw.edu.pl; 2 Department of Pharmacology, Institute of Psychiatry and Neurology, Sobieskiego Street 9, 02-957 Warsaw, Poland; azakrzewska@ipin.edu.pl

**Keywords:** olanzapine, bioanalysis, sample preparation, instrumental analysis

## Abstract

Schizophrenia is a serious mental disorder that significantly affects the social and professional life of patients, causing distortion of reality and loss of identity and cognitive abilities. Psychopharmacological treatment is an integral part of modern psychiatry, and the introduction of new “atypical” antipsychotic drugs has brought significant progress in the treatment of this disorder. One of these drugs is olanzapine, which has an effective effect on the productive symptoms of schizophrenia while having an almost minimal potential to cause extrapyramidal syndrome. However, its effectiveness is confronted with frequent side effects, referred to as “metabolic disorders”. Therefore, to ensure the effectiveness of treatment and to minimize the side effects caused by olanzapine, it is recommended to monitor the drug level during therapy. This article reviews the bioanalytical methodologies that enable efficient extraction and sensitive analysis of olanzapine. We considered the advantages and disadvantages of different sample pretreatment methods, including traditional and novel strategies. The analytical conditions required for the separation and detection of olanzapine and its metabolites were analyzed using chromatographic methods combined with various detectors.

## 1. Introduction

**Figure 1:**
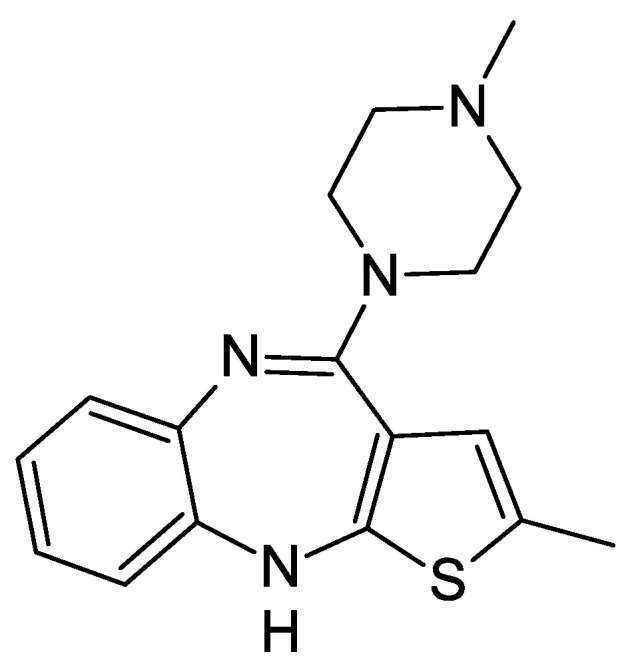
Structural formula of olanzapine.

Olanzapine (2-methyl-4-(4-methyl-1-piperazinyl)-10H-thieno[2,3-b][1,5]benzodiazepine) is a thienobenzodiazepine derivative. Its structure is based on a diazepine ring fused with a thiophene ring and a benzene ring, shown in Figure 1. It is a characteristic yellow substance, occurring in many crystal structures [1]. Olanzapine is practically insoluble in water, but soluble in chloroform. It was developed and introduced to the market in 1996 by the pharmaceutical company Eli Lilly and Company under the name Zyprexa^®^ as an antipsychotic drug used to treat schizophrenia. After the expiry of the patent protection period in 2011, olanzapine became a generic drug [2].

**Figure 2:**
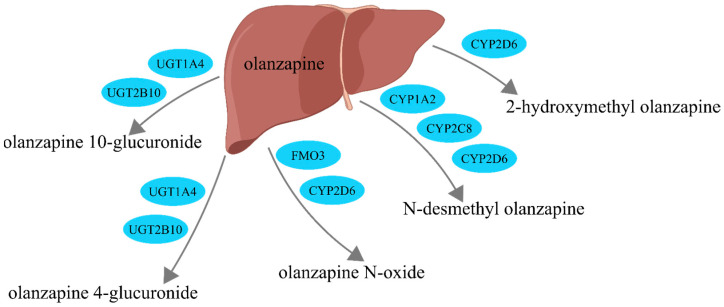
Scheme of olanzapine metabolism in the liver.

It is available in the form of coated tablets with a content of 2 to 20 mg of the drug, as an orally disintegrating lyophilizate in doses of 5 or 10 mg, and as a powder for solutions for injection at a dose of 10 mg [3]. Since olanzapine is administered in doses of 2 to 20 mg daily, its plasma level is relatively low, ranging from 22 to 146 nmol/L [4,5]. Additionally, about 90% of it is bound to plasma proteins [6]. After administration, it reaches its maximum concentration after about 6 h, and the half-life in plasma is over 30 h [7]. The drug is mainly metabolized in the liver to 10- and 4′-N-glucuronides, 4-N-desmethyl olanzapine, olanzapine N-oxide, and 2-hydroxymethyl olanzapine [4,6] (Figure 2). Flavin-containing monooxygenase 3 (FMO3) and enzymes that belong to the group of cytochrome P450 enzymes (CYP1A2, CYP2D6, CYP2C8) are phase I enzymes responsible for the oxidation process; UGT1A4 and UGT2B10 are phase II enzymes of drug metabolism responsible for the coupling process (glucuronidation). UGT1A4 and UGT2B10 are enzymes that transform small lipophilic molecules, such as drugs, steroids, hormones, and bilirubin, into water-soluble and excretable metabolites. UGT2B10 exhibits higher affinity than UGT1A4.

Studies have shown that olanzapine has affinity for serotonin 5-HT_2A/C_, dopamine D_1–4_, histamine H_1_, α_1_ adrenergic, and muscarinic M_1–5_ receptors [4,8,9]. It has an effective action profile in relation to the positive symptoms of psychoses (agitation, aggression, hallucinations, and delusions) and their negative symptoms (social withdrawal, lack of motivation). It has a significant effect on reduced cognitive functions (short-term memory impairment, difficulties in fluent speech, working memory disorders), and at the same time has an almost minimal potential to cause adverse extrapyramidal effects (dystonias, parkinsonism, akathisia) [10]. However, there are significant side effects of taking olanzapine, such as weight gain, leading to obesity, hypertension, insulin resistance, hyperglycemia, dyslipidemia, and type II diabetes [11]. The development of metabolic syndromes often leads to discontinuation of pharmacotherapy by the patient and increases the risk of recurrence of the disease [12]. Therefore, to ensure the effectiveness of treatment and to minimize the side effects caused by olanzapine, it is recommended to monitor drug levels during therapy. Controlling the concentration of the drug and its metabolites allows us to determine the correctness of the adopted treatment strategy, the metabolism of the parent compound, and whether the patient is taking the drug as prescribed. Bioanalytical methods for the determination of olanzapine and its metabolites’ concentrations in biological material, apart from therapeutic monitoring of drug levels, also include pharmacokinetic studies, bioavailability and bioequivalence studies, and toxicology studies. This review article summarizes the published analytical methods used in the analysis of olanzapine in various biological matrices.

## 2. Matrix

In most cases of bioanalytical methods, olanzapine is determined in plasma, and less frequently in serum and whole blood. L. Patteet et al. tried to use an alternative sampling technique such as dry blood spots. However, olanzapine and its metabolite 4-N-desmethyl olanzapine were rejected from the final method, probably due to their instability [13]. However, in their work, C. Ruggiero and co-workers presented a method for the therapeutic monitoring of olanzapine in samples of dry plasma spots [14]. However, this alternative research material does not preclude the need for venous blood sampling, which for a patient with mental disorders is often perceived as unpleasant and even terrifying. M. Josefsson’s team used cerebrospinal fluid in addition to serum to determine olanzapine concentrations. Cerebrospinal fluid is a rare matrix due to collection via lumbar puncture, which is a very invasive procedure and requires trained medical personnel [15]. However, the analysis of brain tissue to quantify olanzapine in forensic cases is intended to assist in the interpretation of potential suicide. Because even after short-term pharmacological treatment, antipsychotic drugs reach high concentrations in the brain [15]. Nevertheless, in recent years, alternative materials such as urine, saliva, and hair have become a subject of considerable interest [16]. The advantage of such material is, above all, the non-invasive method of its collection, thanks to which it does not require the presence of specialized medical personnel and evokes significantly less resistance on the part of the patient. In addition, conventional biological matrices such as plasma, serum, or whole blood reflect drug exposure for only a short period after drug administration. In this case, biological material such as hair allows retrospective control of compliance with medical recommendations [17]. Additionally, saliva is a convenient alternative material for urine, in which there may be a suspicion of metabolic adulteration of the active substance [18]. This may occur as a result of back conversion of the metabolite to the parent drug by potentially unstable metabolites such as glucuronide derivatives and olanzapine N-oxide. However, the saliva sample itself may be difficult to collect due to dry mouth caused by olanzapine use [19]. In this case, stimulation of salivation during sample collection may lead to false drug levels due to salivary flow and reduction in the pH gradient between saliva and plasma [18]. Therefore, it should be remembered that the type of material, and thus the matrix, is of key importance in the development of the analytical method.

## 3. Sample Preparation

**Figure 3:**
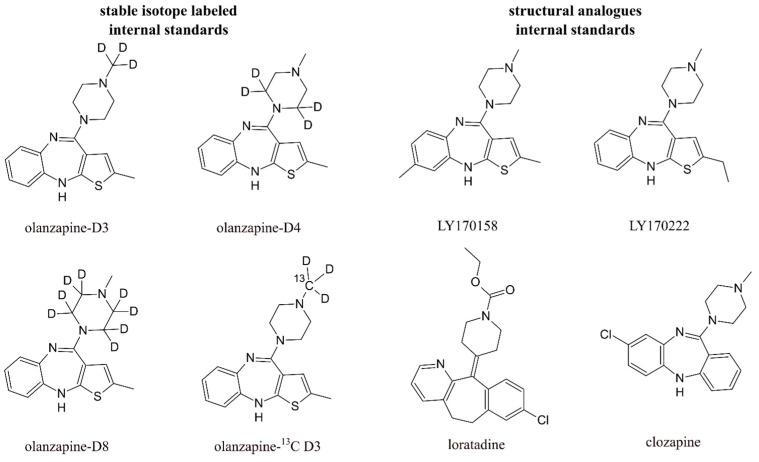
Structural formulas of selected internal standards in the analysis of olanzapine.

Due to the low level of the drug in the biological material caused by the administration of low doses and the reduced bioavailability of the active substance, it is necessary to purify and concentrate the analyzed compound before performing the final detection in order to strengthen the signal and increase the quantification of the method. Typical biological sample preparation methods such as protein precipitation (PPT), liquid–liquid extraction (LLE), and solid-phase extraction (SPE) are among the most commonly used sample cleanup techniques for biological sample analysis. However, there is a growing number of studies involving innovative approaches to sample preparation, such as supported liquid extraction (SLE), Hybrid SPE–Precipitation technology, Dispersive Pipette XTRaction (DPX), or micro extraction by packed sorbent (MEPS). There have also been attempts to automate the preparation of biological samples by using on-line SPE with column switching. As the preparation process additionally involves dilution, evaporation, and reconstitution, there are inevitable variable losses of the analyte. Moreover, in the case of instrumental analysis, there may be changes in signal acquisition (attenuation or amplification) caused by co-eluted components of the matrix. Thus, the actual recovery of the analyte must be determined during method development; otherwise, the results obtained will be of questionable quality. The most common practice for determining recovery is to add an internal standard (IS) to the matrix at the very beginning of the analysis [20]. When working with biological matrices, it is important that the selected internal standard is characterized by a similar strength of binding to proteins and other components of the matrix. It should also have comparable physicochemical properties and therefore behave like olanzapine and its metabolites during sample pretreatment. At the same time, the properties of the internal standard must be unique enough to clearly distinguish it from the analyte at the stage of quantification. There are two types of internal standards (Figure 3). One of them is compounds labeled with stable isotopes, in the structure of which several atoms are replaced by their isotopic equivalents, for example, ^1^H for D, ^14^N for ^15^N, or ^12^C for ^13^C. Olanzapine analysis uses labeling with 3, 4, or 8 hydrogen isotopes. The second type of internal standard is structural analogs. It is recommended that the core structures of the analyzed compounds are based on the same groups and differ only in the length or position of the aliphatic groups. Group modifications that change the functionality of the compound (e.g., -COOH, -SO_2_, -NH_2_, halides, heteroatoms) lead to significant differences in recovery during extraction and, in the case of mass spectrometry analysis, significant differences in ionization efficiency between the analyte and internal standard. When quantifying olanzapine using structural analogs as an internal standard, olanzapine with an additional methyl group on the benzene ring (LY170158), olanzapine ethyl analog (LY170222), or other atypical antipsychotics are used, among others. In the last example, it must be ensured that the selected drug as a standard is not used in therapy in conjunction with the analyte and is not present in the sample. Studies have shown that methods using internal standards labeled with stable isotopes are characterized by better linearity, precision, and accuracy. They better correct analyte losses during the various stages of sample preparation than internal standards that are structural analogs [20,21,22].

### 3.1. Protein Precipitation (PPT)

**Figure 4:**
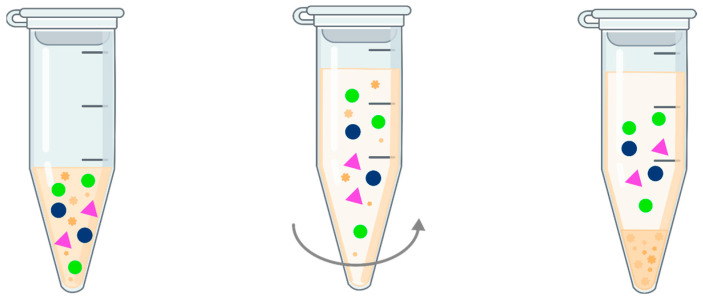
Scheme of protein precipitation; 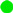 olanzapine, 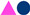 matrix components, 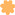 proteins.

Protein precipitation is the oldest and most basic method of preparing biological material with a high-protein matrix (whole blood, plasma, serum). Acid solutions, organic solvents, and metal salts are most often used as precipitating agents [23]. Among the protein precipitation methods used in the preparation of olanzapine-containing samples, the precipitating agent used was an organic solvent miscible with water in a volume three or four times that of the sample tested. Adding a solvent such as acetonitrile or methanol lowers the dielectric constant of the protein-containing solution, which increases the attraction between charged particles and facilitates their electrostatic interaction. This leads to the displacement of water from the hydrophobic surface area of the proteins, which in turn leads to the breaking of the hydrophobic interactions between them, thus causing the proteins to precipitate out of the solution (Figure 4) [24]. Y. Cao et al. (2020) proposed a basic procedure for sample preparation by protein precipitation. Methanol was added to the serum and internal standard solution (serum-to-precipitant ratio 1:3). After vortex mixing, the samples were centrifuged to separate the precipitated protein, and the clear supernatant was subjected to instrumental analysis by HPLC-MS/MS [25]. On the other hand, N. Ansermot et al. (2013) added a solution of an internal standard and acetonitrile to a plasma sample (plasma-to-precipitant ratio 1:3). The mixture was vortexed, then placed in an ultrasonic bath and centrifuged to remove precipitated protein. To concentrate the obtained supernatant, it was evaporated to dryness and reconstituted in 10 mM ammonium formate aqueous solution/acetonitrile (85:15). The solution was mixed and re-centrifuged to remove insoluble compounds, then analyzed by UHPLC-MS/MS [26]. The incorporation of an internal standard into the precipitating solvent to reduce the number of pipetting steps is a common practice, used by H. Lou et al. (2015), where they added a solution of the internal standard in methanol to the plasma (plasma-to-precipitant ratio 1:4). After mixing and centrifugation of the sample, the clear supernatant was analyzed by HPLC-MS/MS [27]. However, when the analyte is largely bound to proteins, one of the concerns with this solution is the possibility of a more variable recovery of the analyte compared to the internal standard [28]. The advantages of protein precipitation are simplicity, minimal sample loss, inexpensive reagents, relatively little labor, and ease of automation, which was used by the team of T. N. Andreassen et al. (2015). Automated sample preparation was performed with a Tecan Freedom Evo pipetting robot (Tecan Nordic, Mölndal, Sweden) that added an internal standard and 1% formic acid in frozen acetonitrile to the serum (1:3 ratio of serum to precipitant). After mixing, the samples were filtered to remove precipitated proteins and subjected to instrumental analysis using UPLC-MS/MS (Waters) [29]. The disadvantages of protein precipitation are dilution of the analyte, and other components of the matrix such as lipids, phospholipids, and salts will remain in solution. These can be potential causes of problems with the chromatographic column, such as reduced column life, elevated baseline signal, shifts in analyte retention time, or loss of selectivity through signal attenuation or amplification, thereby leading to inconsistencies or inaccuracies in the detection and quantification of olanzapine and its metabolites.

### 3.2. Liquid–Liquid Extraction (LLE)

**Figure 5:**
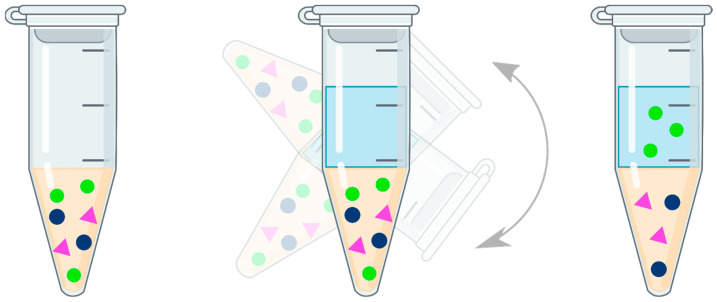
Scheme of liquid–liquid extraction; 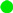 olanzapine, 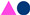 matrix components.

Liquid–liquid extraction is a classic method of isolating drugs from biological samples, using the phenomenon of substance partitioning between two immiscible phases, which are usually formed by water and an organic solvent. Based on the difference in solubility of the analyte in the two solvents, the non-polar compounds migrate to the organic phase, leaving the polar molecules in the aqueous phase (Figure 5) [30]. Extraction efficiency can be modified by adjusting the pH and ionic strength in the aqueous phase. By conducting the process at a pH in which the analyte is in a non-ionized form, its migration to the organic phase is facilitated [31]. For example, the pKa value of olanzapine is greater than 7, so the non-ionized form of the compound occurs at pH > 7. Teams using liquid–liquid extraction during research, after adding an internal standard to the test sample, alkalized it with sodium hydroxide [32,33,34,35,36,37,38], ammonium formate [39,40], carbonate buffer [41,42], sodium carbonate [43,44,45,46,47], disodium phosphate [48], and TRIS buffer [49,50]. Then, the selection of the appropriate solvent is a key step in optimizing the liquid–liquid extraction; important aspects in the selection of solvents for the extraction procedure are the polarity of the analytes and their ability to bind to proteins. To extract the analyte quantitatively, the solvent chosen should not only dissolve olanzapine completely but also be able to break the association with proteins. In the methodology of preparing samples containing olanzapine, the following were used: ethyl acetate [32,47], diethyl ether [43], isopropyl ether [48], and tert-butyl methyl ether (MTBE) [19,33,40,41,49,51,52]. Mixtures of organic solvents were also used, the extraction properties of which may be better than for each solvent alone: hexane/MTBE [39], hexane/dichloromethane [44], hexane/isoamyl alcohol [37], heptane/isoamyl alcohol [36,42], diethyl ether/diisopropyl ether [53], diethyl ether/dichloromethane [34,54], butyl acetate/butanol [50], butanol/cyclohexane [45], and dichloromethane/cyclohexane [46]. Shaking the samples to increase the contact surface of the two phases allows for more effective migration of the analyte to the organic phase. Phase separation is most often carried out by centrifugation or freezing of the aqueous phase. The organic phase can be subjected to instrumental analysis immediately after separation, or dried under a stream of nitrogen in order to concentrate the analytes. Then, the sample is subjected to instrumental analysis after reconstitution. The advantage of LLE is the low cost of materials, the possibility of analyte concentration, and matrix purification, including very effective removal of phospholipids. The main disadvantages of LLE are the complexity of the process, its time-consuming nature, and the requirement to use large amounts of toxic solvents, which pose a potential threat to human health and the environment [28]. In addition, handling biological samples rich in surfactant-like compounds (phospholipids, proteins) can make phase separation problematic by forming emulsions that may prevent further analysis [55]. The limited compatibility of extraction solvents with plastic vessels is another problem that makes glass one of the few possible extraction materials.

### 3.3. Supported Liquid Extraction (SLE)

**Figure 6:**
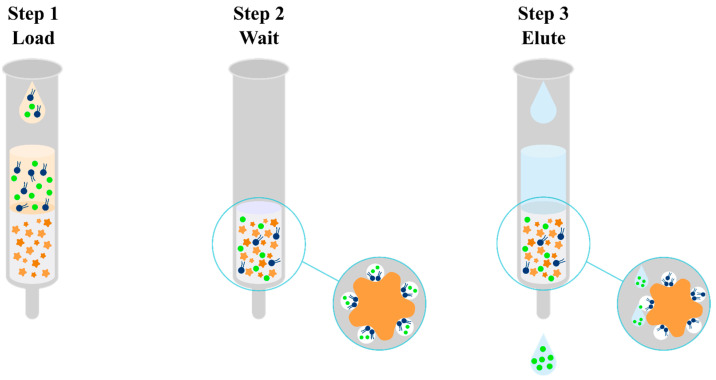
Scheme of supported liquid extraction; 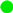 olanzapine, 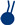 matrix components, 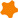 diatoms.

Supported liquid extraction is another drug isolation method that has found application in the preparation of biological samples containing olanzapine. It is an extraction technique that uses the phenomenon of partitioning a substance between two immiscible phases, as in conventional liquid–liquid extraction. The aqueous sample, which is usually plasma [56,57,58] or whole blood [56,59], is pretreated so that the analyte is in a non-ionized form, facilitating its migration to the organic phase. Teams using supported liquid extraction during tests for the determination of olanzapine and its metabolites, after adding an internal standard to the test sample, alkalized it with ammonium acetate [56]. The aqueous sample is then applied and held in place by a highly polar solid carrier. This carrier is purified, chemically inert, highly porous diatomaceous earth (SiO_2_). During this time, the sample soaks into the substrate, and its components concentrate on the surface of the carrier, which results in the subsequent increase in the contact area of the two phases, allowing for effective migration of the analyte to the organic phase. The sorbent bed-wetting process usually takes several minutes. The material is then washed with a non-polar organic solvent, eluting the analytes of interest and leaving undesirable matrices such as salts and phospholipids on the surface of the sorbent bed (because they are not soluble in common organic solvents and do not pass into the final extract). Solvents used in bioanalytical methods of olanzapine determination include dichloromethane [56,57] and ethyl acetate [58]. Studies have shown that the use of dichloromethane, methyl tert-butyl ether, and ethyl acetate effectively removes phospholipids from the analyzed sample, while organic solvents miscible with water, such as acetonitrile and methanol, hinder their removal, leading to increased matrix effects [60]. The entire SLE process can be carried out under the influence of gravity or with the use of positive pressure during sample application and solvent recovery during elution. The undoubted advantages of supported liquid–liquid extraction include a simple and quick “load–wait–elute” procedure (Figure 6), no emulsion formation, extracts free of proteins and phospholipids, lower consumption of organic solvents, and ease of automation.

### 3.4. Solid-Phase Extraction (SPE)

**Figure 7:**
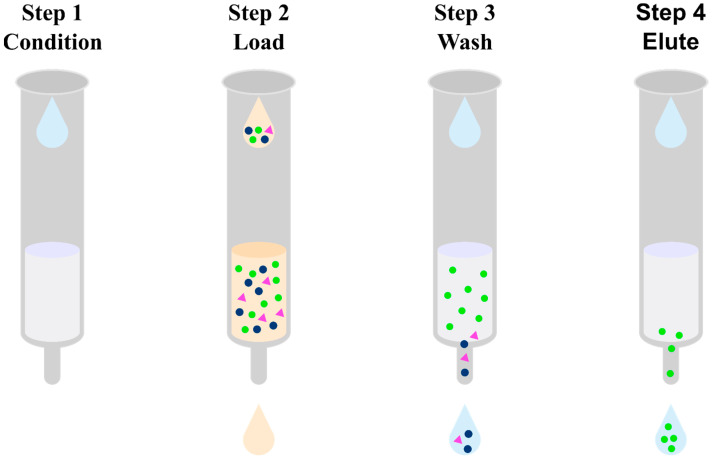
Scheme of solid-phase extraction; 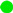 olanzapine, 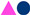 matrix components.

Solid-phase extraction is a method of isolating an analyte in a liquid–solid system, using the phenomenon of analyte partitioning between a liquid sample and a solid, properly selected sorbent (Figure 7) [61]. For the preparation of biological samples containing olanzapine and its metabolites, among the many SPE sorbents available on the market, the following have been used: non-polar C_8_ or C_18_ alkyl groups [62,63,64,65,66,67], a hydrophilic modified styrene-based polymer characterized by a hydrophilic–lipophilic balance (HLB) [68,69,70,71,72,73], and a mixed mode based on non-polar groups and a strong cation exchanger such as benzenesulfonic acid with a counterion which is a strong cation exchanger due to its very low pKa (<1.0) (MCX) [74,75,76,77,78,79,80,81,82]. In the case of SPE using a non-polar medium using a reverse-phase mechanism, olanzapine should initially be converted to a non-ionized form to increase its affinity for the sorbent. For this purpose, a 2 mM ammonium acetate buffer at pH 8.5 [66] and 0.01 M ammonium carbonate at pH 9.3 [65] are added to the test sample with the addition of an internal standard. The test sample is applied to SPE cartridges conditioned with methanol and water [62], which are then cleaned of polar impurities. For this purpose, a solvent with a low elution power is used, such as water [62] or a 5% solution of methanol in water [66]. The analyte is then eluted through a solvent mixture with a higher elution strength and often acidified to re-ionize olanzapine, thereby reducing its affinity for the non-polar sorbent. For this purpose, for example, 5% formic acid in acetonitrile [66] or 0.5 M acetic acid and methanol [65] are used. When a polymer matrix with lipophilic and hydrophilic functional groups (HLB) is used, the retention mechanism is also mainly based on reverse-phase interaction. Thanks to the modification of the hydrophilic stationary phase, it is additionally selective for more polar compounds. The SPE procedure using a mixed mode based on a bed containing both non-polar groups and a strong cation exchanger uses two mechanisms of a compound retention–reversed-phase system, implemented by non-polar groups of the sorbent, and ion exchange, implemented by benzenesulfonic acid. A mixture of methanol and water [77] but also methanol and phosphate buffer with pH 6 [75], methanol and 0.1 N hydrochloric acid [81], and 1 M citric acid solution [78] are used to condition such a sorbent. Then, samples with the addition of an internal standard should be acidified by adding a 2% formic acid solution [77], a 1 M citric acid solution [78], a 0.1 N hydrochloric acid solution [81], or a 25% orthophosphoric acid solution [73]. Lowering the pH of the solution will allow the transfer of ionized olanzapine to the form to increase its affinity for the sorbent. The matrix is washed by a mixture of methanol and water [69], methanol and 1M citric acid solution [78], methanol and 2% formic acid solution [77], or methanol and 0.1 N hydrochloric acid solution [81]. The elution of the analyte takes place at a higher pH in order to convert olanzapine to the non-ionized form and thus reduce its affinity for the sorbent. For this purpose, 2% ammonium hydroxide in ethyl acetate [75] or 5% ammonia solution in methanol [77] is used. Regardless of the sorbent used, the eluted fraction is often evaporated and reconstituted in a smaller volume of solvent prior to instrumental analysis. An undoubted advantage of extraction into the solid phase is the purification of the sample from impurities while concentrating it. This makes it possible to reduce the steps involved in sample preparation, which reduces the number of errors. In addition, solid-phase extraction can be fully automated. Despite all the advantages listed above, SPE is time consuming and relatively expensive as the SPE cartridges produced are disposable. They sometimes have poor batch-to-batch reproducibility and still require relatively large amounts of organic solvents. Nevertheless, SPE is the most frequently used technique for the preparation of biological samples containing olanzapine and its metabolites.

### 3.5. Micro Extraction by Packed *Sorbent*

**Figure 8:**
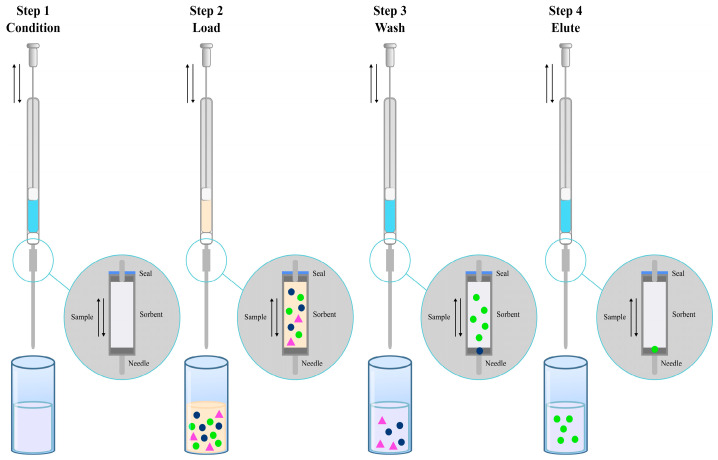
Scheme of micro extraction packed sorbent; 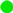 olanzapine, 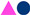 matrix components.

Micro extraction by packed sorbent (MEPS) is a miniaturization of conventional SPE and has also been used during the preparation of biological samples containing olanzapine by the team of S. Hendrickx (2016) [83]. The analyte isolation kit for this liquid–solid system consists of a 100–250 μL microsyringe with a replaceable needle equipped with a small container filled with a small amount of sorbent (Figure 8). This technique combines sample extraction, pre-concentration, and clean-up in a single device. The full device can be operated in different ways, from manually to on-line. The MEPS approach to sample preparation is suitable for reversed phases, normal phases, mixed mode, or ion exchange chemistries. The same types of sorbents are used as in conventional SPE, and the team of S. Hendrickx used a mixed mode based on non-polar groups and a strong cation exchanger at an amount of 4 mg. The MEPS procedure is analogous to the previously described SPE. The first stage is the conditioning of the sorption bed, in this case with a 5% aqueous solution of ammonia and MeOH (20:80) and MeOH, then with a mixture of Ringer’s solution and phosphate buffer (0.833 M, pH 2.5) (1:3). The biological samples should then be acidified by adding a phosphate buffer at pH 2.5 to convert olanzapine to its ionized form and increase its affinity for the sorbent. The sample is adsorbed on the bed as the syringe is inserted and emptied, so this process is repeated several times. Then, the matrix is washed with 5% acetic acid and a mixture of methanol and water (10:90). The elution of the analyte was carried out at a higher pH to convert olanzapine to the non-ionized form and thus reduced its affinity for the sorbent, using a mixture of 5% aqueous ammonia and MeOH (20:80). The obtained extract, before entering the cap-LC-UV system, was diluted with 10 mM ammonium acetate with the addition of 0.05% triethylamine in order to reduce the methanol fraction. S. Hendrickx’s team performed all micro extraction steps manually. MEPS allows for a significant improvement in analytical activities by reducing sample preparation time to a few minutes compared to conventional SPE methodology, allows easy automation, reduces solvent consumption, and makes multiple uses of the syringe with the deposit possible, as opposed to disposable SPE columns, which has a real impact to reduce analysis costs [84]. A problematic aspect of MEPS sample preparation may be optimizing the piston speed, which is crucial for analyte recovery. This is because too high a speed of movements prevents adsorption of the analyte on the MEPS support and can lead to misleading recovery and reproducibility results.

### 3.6. Dispersive Pipette XTRaction (DPX)

**Figure 9:**
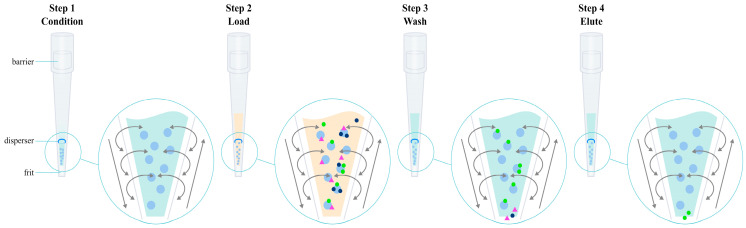
Scheme of Dispersive Pipette XTRaction; 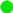 olanzapine, 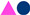 matrix components, 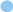 loose resin.

The Dispersive Pipette XTRaction (DPX) technique is a modification of the conventional SPE, which has become an interesting alternative for the preparation of biological samples containing olanzapine. DPX and SPE are based on the same separation principles promoted by the affinity of the analyte to the stationary phase. The kit to carry it out is quite simple and consists of commercially available 1 mL or 5 mL automatic pipette tips, containing loosely contained sorbent material trapped between two filters, the lower and the upper (Figure 9). The team of V. Samanidou (2013) extracted analytes and interfering compounds contained in the matrix after introducing them into a cartridge containing a non-polar sorbent enabling extraction in the reversed-phase system [85]. In the conditioning stage, MeOH and then H_2_O were used to activate the sorbent sites, enabling the appropriate molecular interaction of the solid phase and olanzapine. After conditioning, the alkalized urine sample was aspirated with air to accelerate mixing and kept in contact with the sorbent. The method of distributing the sorbent in the pipette tip enables dynamic mixing with the solvent, which leads to a fast adsorption equilibrium between the solid phase and the analyte [86,87]. Control of the mixing time allows for the proper chemical interaction through van der Waals forces, reaching the equilibrium time for olanzapine adsorption in the sorbent particles, and consequently ensuring efficient extraction and good reproducibility. After the time needed to establish the dynamic equilibrium of the analytes and the sorbent, the sample is discarded from the tip. The next step consists of a washing step in which H_2_O is used to remove interfering substances. The last step is the elution of the analytes using ACN. Olanzapine desorbs from the sorbent and migrates to the liquid phase. The development of the Dispersive Pipette XTRaction (DPX) has contributed to a significant reduction in the necessary volume of the test sample and solvents; as a result, it generates less waste, is safer for laboratory workers, and is also less labor intensive [87].

### 3.7. Hybrid SPE–Precipitation (Hybrid SPE-PPT)

**Figure 10:**
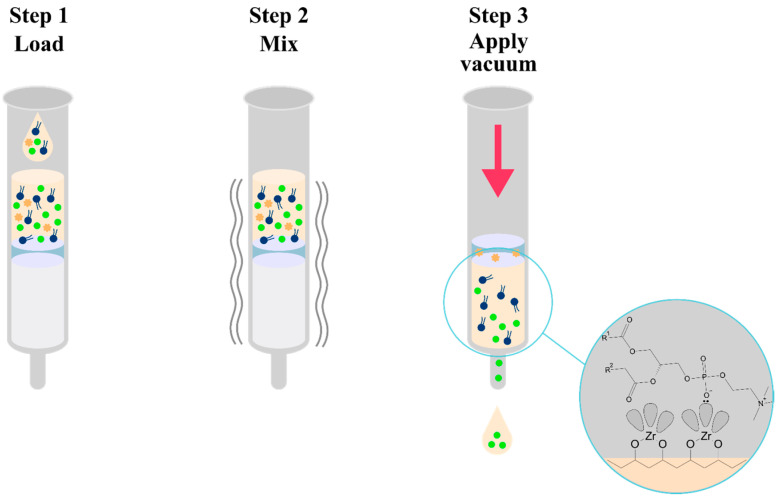
Scheme of Hybrid SPE–Precipitation; 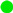 olanzapine, 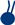 phospholipids, 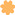 proteins.

Due to the inherent chemical nature of phospholipids, which are a hydrophobic tail and a zwitterionic polar head, they are often co-extracted with analytes of interest during the sample preparation process. To avoid this, an interference removal strategy was developed using Hybrid SPE–Precipitation technology that combines the simplicity of protein precipitation and the selectivity of solid-phase extraction (SPE). This technology uses a silica bed covered with zirconia, which has a selective affinity for phospholipids while remaining non-selective towards a number of basic, acidic, and neutral compounds. The mechanism of phospholipid retention is based on the highly selective Lewis acid–base interaction. Zirconia exhibits properties of a Lewis acid (electron acceptor) which will interact strongly with a Lewis base (electron donor). The phosphate group present in all polar heads of phospholipids acts as a very strong Lewis base (Figure 10) [88]. The team of M. C. Sampedro (2012) used Hybrid SPE-PPT technology to prepare homogenized brain samples [89]. In the first step, the brain tissue was subjected to protein precipitation by adding acetonitrile with formic acid. After mixing and applying a vacuum, the Hybrid SPE-PPT cartridge acts as a filter to remove endogenous substances from the sample. Small molecules such as olanzapine pass through the system uninterrupted. The obtained filtrate is free of proteins and phospholipids, ready for LC-MS/MS analysis. Nevertheless, M. C. Sampedro’s team evaporated the eluate to dryness under a stream of nitrogen and reconstituted it in a mixture of ammonium formate (pH 8.2) and ACN.

### 3.8. On-Line SPE

**Figure 11:**
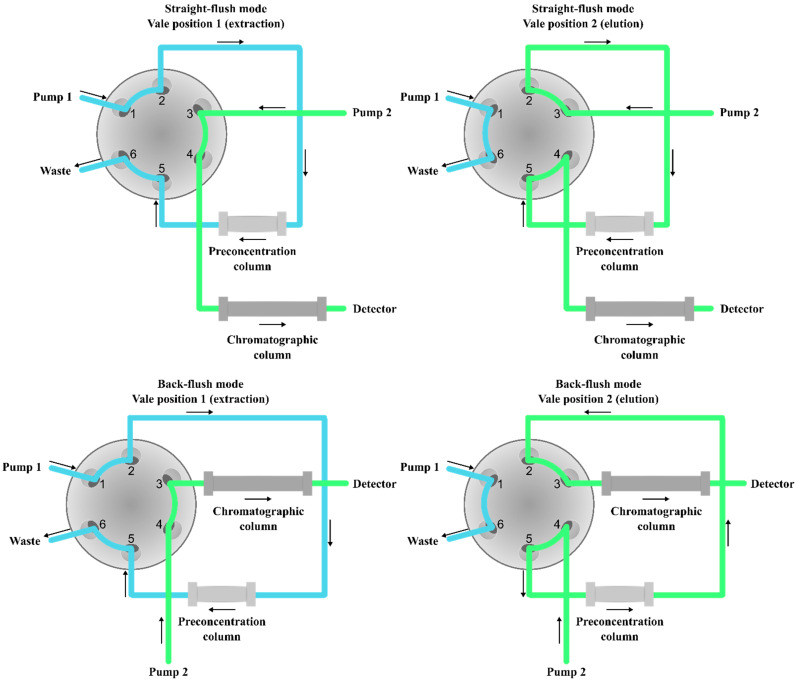
Graphical diagrams of the column-switching system in the straight-flush and back-flush modes.

Conventional methods for the preparation of olanzapine and its metabolites containing biological samples require manual multi-step preparation procedures, which are time consuming and subject to errors and loss of reproducibility. The solution to these problems may be sample preparation in an automated analytical process performed by off-line or on-line workstations. In the off-line mode, samples prepared by the workstation must be independently removed and independently injected into the analyzer, which was the case with the previously mentioned team of T. N. Andreassen (2015) during automatic serum deproteinization carried out using a Tecan Freedom Evo pipetting robot (Tecan Nordic, Mölndal, Sweden) and instrumental analysis using UPLC-MS/MS [29]. However, in the on-line mode, the workstation is also used to inject the prepared samples into the analyzer and start data acquisition [90,91,92,93,94,95]. Using the column-switching approach, the SPE sample preparation technique can be easily combined with a chromatography instrument to provide such an on-line system. This system consists of two high-pressure pumps, an automatic six-port valve, a pre-concentration column, and an analytical column [96]. On-line SPE cartridges are commonly called pre-columns because the first cartridges used were the same pre-columns used in HPLC to protect analytical columns [97]. For the preparation of biological samples containing olanzapine, various media packed in stainless steel pre-columns were used, such as porous silica with C_8_ alkyl groups [91,94] or cyanopropyl groups [90], a polymer sorbent with lipophilic and hydrophilic functional groups (HLB) [93], or restricted access material (RAM) [92,95]. RAM sorbents are a class of materials that are able to fractionate a biological sample into a protein matrix and analyte, based on the molecular weight cutoff [15,98]. Fractionation and extraction are based on the simultaneous performance of two chromatographic processes: size exclusion chromatography and reverse-phase chromatography. When a protein-containing sample is injected into the column, the proteins are not retained but eluted immediately on a size exclusion basis because of the sorbent bed’s small pores and because the hydrophilic surface does not allow it to be retained in the column. Low-molecular-weight compounds such as olanzapine are retained and eluted by interaction with the matrix. The first step of on-line SPE is the direct introduction of the sample to the conditioned column, where the analytes are isolated from the other components of the sample matrix and pre-concentrated. Analytes of interest, such as olanzapine, are retained by different mechanisms depending on the type of extraction column used, while other undesirable components of the matrix are leached out and lost [99]. Then, using a six-port valve, the flow direction of the mobile phase is changed to the analytical column, where the chromatographic separation of the analyzed compounds takes place. Elution can be carried out in two different ways, as shown in Figure 11 [96,100,101]. In the straight-flush mode, the mobile phase flows in the same direction as in the purge step [93], while in the back-flush mode, the mobile phase flows in the opposite direction to the purge step [91,92,93,95]. In practice, the back-flush mode is more often chosen because, in this configuration, the endogenous components absorbed at the beginning of the purification column are directed to the waste, thus minimizing the matrix effect, and highly retained analytes are easily transferred to the analytical column in the elution step [96]. In addition, as an alternative to the column-switching approach, there are also several fully automated on-line SPE devices on the market, such as the ASPEC XL (Gilson, Middleton, WI, USA) used by the team of O. V. Olesen (2001), which in this instance allowed direct connection to the HPLC-UV system [102]. Fully automated sample preparation reduces sample preparation time, thereby significantly improving overall laboratory throughput for routine analysis. This solution can reduce the risk of sample contamination and improve accuracy. Additionally, it reduces the risk of operator exposure to infectious materials or toxic solvents [103]. On the other hand, this solution also has disadvantages, such as requiring expensive equipment or low portability.

## 4. Separation Methods and Detection

The main separation technique used in the bioanalysis of antipsychotics and their metabolites is liquid chromatography (LC) [104,105]. Among LC techniques, high-performance liquid chromatography (HPLC) and ultra-high-performance liquid chromatography (UHPLC) are the two dominant separation methods. Both techniques are based on different types of analyte detection, such as electrochemical detection (ECD), ultraviolet detection (UV), and diode array detection (DAD). The most commonly used detection modes are mass spectrometry (MS) and tandem mass spectrometry (MS/MS). Gas chromatography (GC) is much less frequently used for the determination of olanzapine and its metabolites in biological matrices. It is combined with a nitrogen phosphorus detector (NPD), a mass spectrometer (MS), and a tandem mass spectrometer (MS/MS).

### 4.1. Liquid Chromatography (LC)

Given the chemical and physical properties of olanzapine, such as hydrophobicity (octanol–water partition coefficient, logP > 4) and limited volatility (high boiling point 476 °C), it is a good candidate for reverse-phase separation. This system consists of a non-polar stationary phase, which is most often silica chemically bound by covalent bonds with C_8_ alkyl groups [38,39,41,46,48,63,70,71,72,73,74,75,76,77,78,79] or C_18_ [14,19,25,26,27,29,32,33,34,35,44,45,47,48,49,51,52,53,54,57,58,59,63,64,65,66,67,70,71,73,77,79,80,81,82,83,89,90,91,93,94,95,106,107,108,109,110,111,112,113,114,115], and a moderately polar mobile phase consisting of a mixture of water and a polar organic solvent such as methanol or acetonitrile. However, under these conditions, olanzapine exists both as an ionic and undissociated molecule. This causes problems in the analysis due to the different mechanisms of interaction of these two forms of the analyte with the surface of the stationary phase. These are hydrophobic interactions of neutral molecules with alkyl ligands of the van der Waals character and ion–ion interactions of ionized olanzapine molecules with silane residues on the surface of the stationary phase. This situation leads to low separation efficiency and asymmetric peaks, ultimately leading to difficulties in the repeatability of the analysis [61]. To avoid this, the suppression of electrolytic dissociation of olanzapine with a buffer with a higher pH [46,74,75,76,79] or the suppression of electrolytic dissociation of silanols with a buffer with a lower pH can be used [19,26,27,37,38,51,62,65,69,71,77,92,106,111,112], as well as the use of ion-pairing reagents as eluent additives such as triethylamine [44,48,68,69,77,83,105] or trifluoroacetic acid [53], which in lower concentrations are used as silanol blockers. The use of special stationary phases is also described, which, due to various chemically bonded functional groups, provide an alternative selectivity for chromatographic separation based on the π-π interaction. These columns include C_18_-PFP (pentafluorophenyl) [72], C_3_-CN (cyanopropyl) [42,43], and C_6_-Ph (phenylhexyl) [78,92]. Another type of separation of antipsychotic drugs and their metabolites, which is more and more often used, is hydrophilic interaction chromatography (HILIC) [40,56]. This system uses polar stationary phases, used in normal-phase chromatography, and mobile phases with a high content of organic solvents, characteristic of reverse-phase chromatography [115]. Any polar stationary phase can be used for the separation of analytes, but most often it is unmodified silica [40,56]. On the other hand, the mobile phases used are mixtures with a high content of organic solvents (>80%), such as acetonitrile [40,56] and methanol, and a low content of water [56] or an aqueous buffer solution with an appropriate pH [40]. As the ACN content in the mobile phase increases, water is adsorbed more strongly on the polar surface of the stationary phase, forming a hydrophilic layer of the eluent. In such a situation, the organic solvent has limited contact with the active surface of the filling, and this happens until the content of the water component does not exceed 20%. Then, water has the ability to displace ACN from access to the surface of the stationary phase, and a specific liquid–liquid extraction system is created. In addition, thanks to water, analytes can be close enough to functional groups of the stationary phase, enabling other types of interactions, such as hydrogen interactions or electrostatic interactions [116]. However, above 20% of the water component in the mobile phase, there are more and more organic components at the surface of the stationary phase [117,118,119].

**Figure 12:**
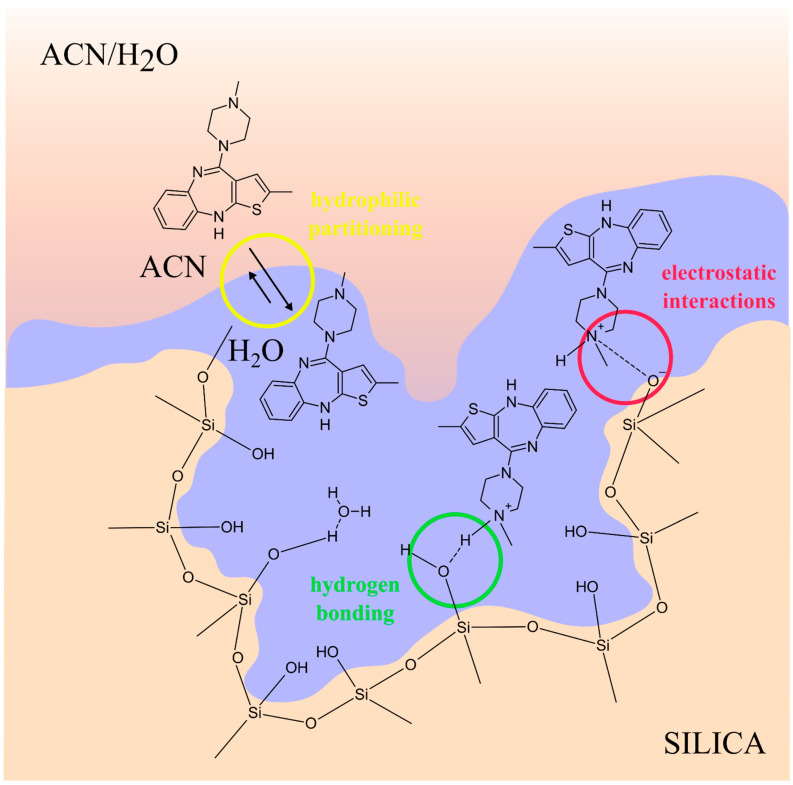
Retention mechanism in HILIC.

Figure 12 schematically shows the mechanism of olanzapine retention on unmodified silica, taking into account three different types of interactions between the stationary phase and the eluent layer adsorbed at its surface. The higher pH of the mobile phase used in the analysis of olanzapine intensifies the ionization of silanol groups on the silica gel surface, increasing the stationary phase’s ability for cation exchange, which can be used during the separation of basic compounds. As a result, analytes are usually strongly retained on the silica gel surface through hydrogen bonds and ion exchange interactions. The symmetry of the peaks of basic compounds separated using the HILIC technique is often better than the symmetry of the peaks for the same compounds separated in the reverse-phase system [120]. The chromatographic columns used in the analysis of olanzapine differ in the diameter, length, and particle size of the stationary phase. Their internal diameter ranges from 2.0 to 4.6 mm, their length from 50 to 250 mm, and grain size from 3 to 5 μm. Such columns are capable of withstanding flowing mobile-phase pressures of the order of 400 bar. However, the construction and marketing of pumps used in ultra-high-performance liquid chromatography (UHPLC), capable of operating at a pressure of 1200 bar, allowed a reduction in the diameter of the stationary-phase grains (<2 μm) [19,26,29,40,51,112,113]. With a decrease in the grain size of the bed, the degree of their packing increases, and thus, the grain surface area per volume unit increases, which results in an increase in the efficiency of the column. Thanks to this, better resolution and shorter time of olanzapine analysis can be achieved [19,26,29,40,51,77,112,114]. The first developed methods of chromatographic separation of olanzapine were achieved by isocratic elution, i.e., with a constant composition of the mobile phase throughout the analysis [32,34,35,36,37,39,40,42,43,44,46,47,50,52,53,54,56,57,62,63,65,66,67,68,69,70,73,74,75,76,90,91,93,94,102,105]. It is a simple and very reproducible method of chromatographic separation. However, it fails when analyzing mixtures containing many chemical compounds with little variation in polarity or molecular weight, as in the case of methods involving many other antipsychotics and their metabolites in addition to olanzapine. Therefore, to separate complex mixtures, gradient elution is used, consisting of a continuous or abrupt change in the composition of the mobile phase during the analysis [14,19,25,26,27,29,33,38,41,45,48,49,51,58,59,64,71,72,77,78,79,80,81,82,92,95,106,107,108,109,110,111,112,113,114].

#### 4.1.1. Liquid Chromatography and Electrochemical Detection (ECD)

Initially, electrochemical detection was used to determine olanzapine concentration due to its extremely low oxidative potential. The principle of operation of the electrochemical detector is based on the oxidation or reduction reaction of the analyzed substance in the flow cell with the participation of the applied constant potential, which leads to the consumption (reduction) or release (oxidation) of electrons at the interface of the electrode. Proposed by J. T. Catlow and colleagues (1995), M. Goodwin and colleagues (1995), J. A. Chiu and colleagues (1996), S. C. Kasper and colleagues (1999), J. Bao and colleagues (2001), and M. L. Lu and colleagues (2013), an electrochemical detector equipped with a series-connected protective cell (e.g., −0.3 V potential) and two analytical cells (e.g., 0.2 V potential in the first, −0.2 V potential in the second) has been used [46,63,74,75,76]. This allowed for high specificity by using a reduction and oxidation mode where compounds in the first assay cell were oxidized at 0.2 V followed by a reduction in the second assay cell at −0.2 V. Monitoring the reduction reaction resulted in greater selectivity because fewer endogenous compounds present in the plasma at this potential were reversibly oxidized. Table 1 presents a comparison of olanzapine determination methods using liquid chromatography with electrochemical detection. Ten studies in the fields of clinical research, pharmacokinetics, therapeutic drug monitoring, and bioequivalence are presented. The determinations were made in human plasma, rat plasma, human milk, and rat brain tissue. LY170222, LY170158, or clozapine was used as an internal standard. The undoubted advantages of using electrochemical delectation include a low detection limit, selectivity, and no need to derivatize the analyte before analysis. However, in complex biological matrices, high voltage causes rapid corrosion of the electrode surface, leading to a decrease in sensitivity. Therefore, sample purification steps are necessary to remove compounds that may lead to contamination of the electrode [121]. In addition, the eluent used in electrochemical detection should be electrically conductive; this can be achieved by adding an auxiliary electrolyte. Using a buffer solution for this purpose is a good choice because it minimizes the influence of the sample matrix and acts as an electrolyte. Phosphate buffer is commonly used in the bioanalysis of olanzapine due to its electrical neutrality [122].

#### 4.1.2. Liquid Chromatography, Ultraviolet Detection (UV), and Diode Array Detection (DAD)

Ultraviolet detection (UV) is an alternative to the rather demanding electrochemical detection when analyzing olanzapine in a biological matrix. Due to the presence of unsaturated bonds and chromoform groups of π-CH=CH- electrons in olanzapine, it is possible to absorb the energy of ultraviolet radiation, which is the result of electronic transitions in the molecule. The UV detector uses a deuterium discharge lamp (D2 lamp) as a light source with a wavelength of light in the range of 190 to 380 nm. The following wavelengths have been used in the bioanalytical methods of olanzapine determination: 214 nm [42], 240 nm [77], 242 nm [92], 254 nm [37,83,90,91], 255 nm [48,107], 260 nm [38], 270 nm [36,44,102], and 280 nm [107]. In recent years, the diode array detector (DAD) has become more and more popular, used in the studies of K. Titier et al. (2003) and E. Dziurkowska et al. (2019). DAD detects radiation absorption in the UV-to-VIS range but differs in its optical system from typical UV-VIS detectors. While the UV-VIS detector has only one light-receiving section on the sample side, the DAD has multiple photodiode arrays to acquire information over a wide range of wavelengths in the same unit of analysis time. Table 2 presents a comparison of olanzapine determination methods using liquid chromatography with spectrophotometric detection. Fourteen studies in the fields of clinical research, bioequivalence and pharmacokinetics, and therapeutic drug monitoring are presented. The analyses were performed in the following matrices: human plasma, serum, urine, saliva, rat plasma, and rat brain tissue. Trifluoperazine, LY170222, fluperlapine, N-desmethyl clozapine, methylrisperidone, clozapine, imipramine, carbamazepine, and chlordiazepoxide were used as internal standards. UV and DAD detectors are known for their stability, versatility, and low maintenance requirements, which have made them very popular for the analysis of antipsychotics (including olanzapine) in biological samples. They are non-destructive techniques that allow serial connection to other detectors, such as mass spectrometry. They are compatible with gradient elution analysis due to their relative resistance to changes in the flow and refractive index of mobile phases and temperature fluctuations. However, it should be remembered that the eluent should not absorb light in the wavelength ranges used for analyte detection. The most popular mobile phases described in the bioanalytical methods of olanzapine using the detection of light absorption are phases based on phosphate buffer or phases with the addition of triethylamine. The phosphate buffer is particularly useful because it can be used at wavelengths less than 220 nm.

#### 4.1.3. Liquid Chromatography Coupled with Mass Spectrometry (MS) and Tandem Mass Spectrometry (MS/MS)

**Figure 13:**
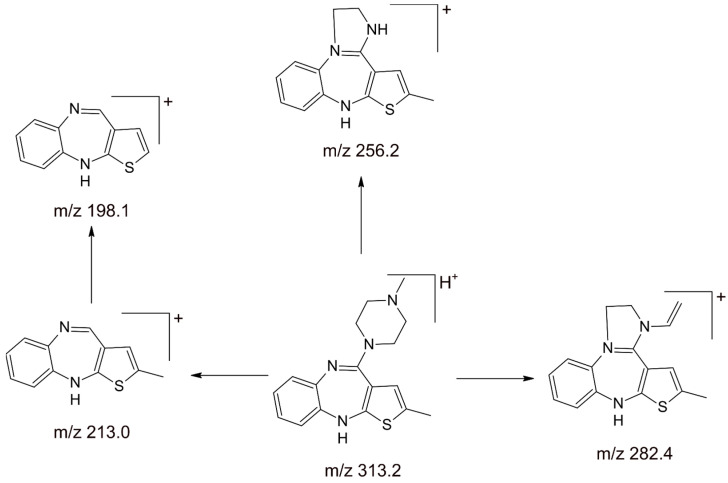
Proposed fragmentation of olanzapine by D. S. Patel [73].

Mass spectrometry imposes requirements on the methods of olanzapine bioanalysis, resulting in a different approach to the development of the analyte separation method. The mobile phase used during the separation of analytes must be volatile because its evaporation is an important step in the analysis. Therefore, so far eagerly used in LC-ECD and LC-UV analyses, phosphate buffer is not suitable for detection by mass spectrometry. It induces ion suppression and gives a high baseline background [123]. Ion suppression leads to an artificial and unreproducible reduction in the olanzapine signal when the constant concentration assay is performed on samples where the background electrolyte concentration changes. The reverse can also occur where the olanzapine response is artificially enhanced, known as ionic potentiation. In addition, the also popular triethylamine is viscous and non-volatile. It causes ion suppression because it has a high affinity for protons in the gas phase. This means that interactions can occur just outside the droplet, in the gas phase, in which triethylamine steals protons from olanzapine and takes away almost all the charge delivered during the ionization process. Triethylamine generates an intense signal at *m*/*z* 102 in positive ion mode, stays in the chromatography system for a long time, and is eluted long after discontinuation of use. Trifluoroacetic acid in high concentrations should also be avoided because, although it is a good source of protons, it has a high propensity to form stable ion pairs with positively charged analytes. The resulting ion pairs in the solution also remain stable during the evaporation process, which ultimately results in significantly reduced ionization sensitivity in the positive ion mode. The use of trifluoroacetic acid results in a very strong signal coming from the *m*/*z* 113 ion in negative ionization in the mass spectrum. Using the LC-MS technique, solvents (or mixtures of solvents) with high volatility and high polarity are used, such as acetonitrile, methanol, 2-propanol, and water. Ammonium formate and ammonium acetate (in the concentration range of 2–10 mM) are used as volatile salts for the preparation of buffers in the olanzapine bioanalysis methods, and formic acid is used as a mobile-phase modifier (in the concentration range of 0.1–1%). Optimization of olanzapine measurement conditions using the LC-MS technique includes optimization of the ion source and interface parameters depending on the conditions of chromatographic analysis, such as flow and composition of the mobile phase, and on the properties of the compound itself. The most common ionization methods used in olanzapine bioanalysis are chemical ionization (APCI) [45,50,51,64,65,72] and electroscattering (ESI) [14,19,25,26,27,29,32,33,34,35,39,40,41,43,48,49,52,53,54,56,57,58,59,66,67,70,71,73,78,79,80,81,82,89,94,95,108,109,110,113,114]. Regardless of the ionization method, olanzapine is detected in the positive ion mode. Then the obtained intensity is higher, because it is basic in nature and able to accept protons, creating pseudomolecular ions of the form [M+H]+. In the APCI technique, the introduced sample is ionized under atmospheric pressure. The sample solution is introduced into the ion source through a capillary coaxially surrounded by the nebulizer capillary. The olanzapine-containing sample is nebulized in a heated pre-chamber at 340 °C to 550 °C by using an atomizing gas of N_2_ at a pressure of 60 psi to 80 psi. In addition, an auxiliary gas (N_2_, from 0.02 L/min to 1.5 L/min) is used, the flow of which minimizes the interaction of the analyte with the capillary wall. If the applied temperature in the heated chamber is set too low, as a result of the short-term exposure to the temperature in the heated chamber, incomplete evaporation of the solvent occurs and large droplets are ejected into the ion source. However, if the temperature is set too high, thermal degradation of the resulting ions occurs. Right next to the capillary outlet, there is a corona discharge electrode, which initiates the olanzapine ionization process at a discharge current of 4 µA to 5 µA. The optimal corona discharge current allows for the best signal-to-noise ratio. The main supplier of cation radicals formed during corona discharges and used for ionization of the analyte is N_2_, present in a large amount in the source space. As a result of corona discharges occurring on the electrode, this gas is intensively ionized, and the obtained highly reactive forms of gas react with solvent molecules, creating secondary ions. After recombination with a neutral solvent molecule, we obtain an ion that reacts with a neutral analyte molecule [124]. In contrast, in the ESI technique, where ionization also takes place at atmospheric pressure, the sample solution is introduced into the ion source through a capillary to which a high voltage of 1500 V to 5500 V is applied. It is recommended to use the lowest possible voltage to avoid corona discharge, which can result in an unstable signal or complete signal loss. In the positive ion mode, the appearance of clusters of protonated solvents, such as H_3_O^+^(H_2_O)_n_ from water and CH_3_OH_2_^+^(CH_3_OH)_n_ from methanol, indicates the presence of a discharge. The introduction capillary is located coaxially in a larger-diameter capillary through which the atomizing gas and the heated drying gas flow. The nebulizing gas in the methods of olanzapine determination is most often N_2_ with a flow of 8 L/min to 16 L/min and a pressure of 10 psi to 60 psi; also, the drying gas is usually N_2_ with a flow of 1.5 L/min to 11 L/min and 20 psi to 70 psi. The presence of a drying gas further helps to focus the stream and increases solvent evaporation. This enables higher flow rates of liquid samples to be handled and increases sensitivity when the heating gas temperature and flow rate cause the solvent to reach a point where it is almost completely evaporated. As the content of organic substances in the mobile phase increases, its optimal temperature decreases. If the temperature of the ion source is set too low, incomplete evaporation occurs and large and visible droplets are ejected into the ion source. If the temperature is set too high, the solvent may evaporate prematurely and the analyte may deposit at the tip of the capillary. The insertion capillary is positioned at 90 degrees to the ion path. Such placement of the source elements protects the inside of the apparatus from solution droplets that do not evaporate completely. Under the influence of the potential difference, the so-called Taylor cone is formed at the outlet of the capillary introducing the sample, which then turns into a very narrow stream (jet). The jet quickly becomes unstable and breaks up into individual droplets, creating a mist of electrically charged droplets. In the next step, droplets of the analyte solution lose their solvent as a result of evaporation. When the repulsive force between the charges of the same ions on the droplet surface becomes greater than the surface tension force that holds the droplet together, it breaks down into smaller droplets as a result of Coulomb decay [124]. Then, for very small drops, the electric field strength on their surfaces is so high that it can cause the emission of single ions into the gas phase. These ions are directed by an electric pile to further parts of the source, and then to the mass analyzer. In order to transfer the ions formed in the APCI or ESI source from the part under atmospheric pressure to the medium-pressure area and finally to the mass analyzer, the potential difference and pressure difference are used. In modern spectrometers, two plates with holes (curtain plate and orifice plate) are used as a link between the atmospheric- and medium-pressure parts. Curtain gas (N2) flows between the plates, which escapes through a larger opening on the atmospheric side (curtain plate) and thus protects the further part of the source against solvent vapors entering it. In addition, the ions collide with inert gas molecules and lose loosely bound solvent molecules. The declustering potential (DP) is the voltage that helps prevent the ions from reaggregating with the solvent. The higher the voltage, the higher the energy transferred to the ions. If the DP parameter is too high, fragmentation may occur. A value that is too low will result in lower ion intensity and potential clustering interference with the solvent. In the methodologies proposed by the olanzapine teams, the potential was optimized for values from 30 to 100 V. Then, the entrance potential (EP) concentrates the ions in the Q0 region. EP is usually set to 10 V for positive ions, has little effect on compound optimization, and generally can be left at default without significantly affecting the detection limits of the analyte. The potential difference between Q0 and Q2 (the collision target) is the collision energy (CE). This is the amount of energy the precursor ions receive as they are accelerated to collide in the collision cell. The higher the collision energy, the more efficient the fragmentation. If the CE is too low, the molecule fragments inefficiently. Too high a CE value may cause the compound to split too much. Collision-activated dissociation (CAD) is the process of collision of precursor ions with an inert gas, which breaks the molecule into fragment ions. The inert gas used in these olanzapine methods is Ar/N_2_ (90:10), Ar, or N_2_. Then, thanks to the collision cell exit potential (CXP), the resulting fragment ions are concentrated, accelerated, and transferred from Q2 (collision cell) to Q3 (the greater the mass, the higher the CXP value). The team of D. S. Patel (2012) proposed the fragmentation of olanzapine presented in Figure 13 [73].

Quantification for tandem mass spectrometers was performed using Multiple Reaction Monitoring (MRM) mode [14,15,19,25,26,29,32,33,34,39,40,41,45,48,49,51,52,54,58,64,66,67,70,71,73,78,79,80,81,82,89,94,95,108,109,110,111,112,114] or Single Reaction Monitoring (SRM) mode [27,50,56,59,72,93], in which the selected ion passes through the Q1 quadrupole and then undergoes fragmentation in the collision chamber. The ion at *m*/*z* 313.2 is a protonated precursor [M+H]+ in Q1 full-scan spectra. The Q3 quadrupole then selects a given fragment ion that reaches the detector. Olanzapine transitions of *m*/*z* 313 → 256, 313 → 213, 313 → 198, and 313 → 169 were observed. The ion at *m*/*z* 256.2 was the most abundant ion in the product ion mass spectra of olanzapine. It resulted from the cleavage of the piperazine ring to CH3NHCH¼CH2 (the neutral fragment). At *m*/*z* 282.4 and 213.0, other characteristic fragments were observed due to the formation of CH3NH2 and the elimination of the piperazine ring. The Q3 quadrupole then selected a given fragment ion that reached the detector. Olanzapine transitions of *m*/*z* 313 → 256, 313 → 213, 313 → 198, and 313 → 169 were observed. Dwell time was set from 50 to 600 ms. The longer the residence time, the greater the number of target ions detected. The interval between observing subsequent MRM passes is the pause time, which is needed to “clean” the ion optics of the spectrometer from ions formed in the fragmentation process. However, in the case of using a mass spectrometer with a single quadrupole analyzer, the selected ion monitoring mode (SIM) [35,43,54,57,65,113] was used for the quantification of the analyte, where monitoring of the intensity of specific *m*/*z* values was set; in the case of olanzapine, it was the [M+H]^+^ 313 ion. Table 3 compares olanzapine methods using liquid chromatography coupled to mass spectrometry or tandem mass spectrometry. Fifty studies in the field of therapeutic drug monitoring, pharmacokinetics, bioavailability, bioequivalence, and toxicology are presented. The analyses were performed in human plasma, serum, whole blood, hemolyzed whole blood, saliva, cerebrospinal fluid, hair, nails, human brain tissue, dry plasma spots, other human body fluids (pericardial fluid, gastric contents, bile, and urine), rat brain tissue, and rat plasma. LY170222, LY170158, dibenzepin, diazepam, diazepam-D5, loratadine, midazolam, remoxipride, duloxetine, quetiapine, haloperidol-D4, venlafaxine, 4-amino-2-methyl-10H -thieno[2,3-b][1,5]-benzodiazepine, diphenhydramine, risperidone, risperidone-D4, anastrozole, repaglinide, topiramate-D12, trimipramine-D3, irbesartan, clozapine, olanzapine-D3, olanzapine-D4, olanzapine-D8, olanzapine-13C-D3, and N- desmethyl olanzapine-D8 were used as internal standards.

### 4.2. Gas Chromatography (GC)

Gas chromatography (GC) is often the technique of choice in bioanalysis, but it is very rarely used for the determination of olanzapine and its metabolites in biological matrices [74,85,125,126,127,128,129,130]. The main reason why it is such a rarely used technique is that olanzapine and its metabolites have limited volatility. The teams of R. M. Goodwin (1993) and J. T. Catlow (1995) transformed olanzapine into a volatile and more thermally stable derivative as a result of a derivatization reaction with heptafluoroacetic acid anhydride [74,125]. However, work on the separation of olaznapine by gas chromatography without derivatization has also been reported [85,126,127,128,129,130]. A properly prepared biological sample is introduced into the GC system through a heated dispenser whose temperature in published works ranges from 200 °C to 310 °C [126,128,130]. Here, compounds in the sample, including solvent components, are heated and evaporated. In most published methods, the entire vapor cloud is transferred to the analytical column by the carrier gas in the splitless mode [129,130]. However, in the case of the methodology proposed by S. Ulrich (2005), only part of the sample is transferred to the analytical column in the so-called split mode (ratio 45:1); the remainder of the vapor is flushed out of the system through the split mode [128]. The mobile phase, called the carrier gas, always flows sequentially from the injector to the column and then to the detector. In the olanzapine assay methods, inert gases such as helium [126,129,130] and nitrogen [128] were used as the mobile phase. Thermostatic non-polar capillary columns such as HP-5 MS [128,130] and DB-5 MS [127,129] were used to separate the sample components in a controlled temperature program. In the column, the mixture of compounds was separated into different components, and then the amount of each compound was measured using a suitable detector.

#### 4.2.1. Gas Chromatography Coupled with Mass Spectrometry (MS) and Tandem Mass Spectrometry (MS/MS)

Gas chromatography coupled with mass spectrometry is the most commonly used analytical platform for bioanalytical studies of olanzapine. This is due to the reproducible and selective nature of the technique, as well as the high availability of spectral libraries [131]. The teams of R. M. Goodwin (1993) and J. T. Catlow (1995) used a soft ionization technique, which was negative chemical ionization, to create ions. Thanks to the derivatization of olanzapine with heptafluoroacetic acid anhydride and the introduction of electronegative fluorine atoms into the structure of the molecule, the analyte was able to capture the electron and stabilize the negative charge [74,125]. In this technique, there is relatively little fragmentation of the analyte and only molecular ions are observed for most molecules. The teams of A. J. Jenkins (1998), T. C. Merrick (2001), K. Ikeda (2012), and T. Rosado (2018) used a hard ionization technique, which was electron ionization [126,127,129,130]. Here, olanzapine molecules are bombarded by electrons emitted from a hot filament. Electron ionization is performed using electrons with a kinetic energy of 70 eV, which leads to the formation of highly reactive radical cations. The excess energy remaining after ionization is dissipated by fragmentation of the analyte. The team of T. Rosado (2018), who used a tandem mass spectrometer, quantified olanzapine using the Multiple Reaction Monitoring (MRM) mode. Transitions of olanzapine of *m*/*z* 301.8 → 285.1 and 301.8 → 286.1 have been observed [130]. However, in the case of using a mass spectrometer with a single quadrupole analyzer, the selected ion monitoring mode (SIM) was used to quantify the analyte, where the intensity of specific *m*/*z* values was monitored (in the case of olanzapine, it was *m*/*z* = 370 (derivatized olanzapine) [74,125]), as well as the SCAN mode, where the full range of mass to charge ratio (*m*/*z*) from 35 to 450 amu was scanned [126,127].

#### 4.2.2. Gas Chromatography with a Nitrogen Phosphorus Detector (NPD)

Due to the presence of nitrogen atoms in the olanzapine molecule, it is possible to use a nitrogen–phosphorus detector (NPD) for its quantitative determination in biological material [85,128]. It is a selective ionizing detector using a hydrogen/air flame, the operating principle of which is based on the variability in the electrical conductivity of the flame. Between the stream leaving the column and the polarizing electrode, there is a bead of rubidium or alkaline cesium chloride in a silica system, mounted on a platinum wire. When heated, it emits electrons, which are collected at the anode level and provide the base current. When olanzapine leaves the column, partially burnt nitrogen compounds are adsorbed on the surface of the bead, which causes an increase in the amount of emitted electrons (and thus the current), which corresponds to the reaction of the detector.

### 4.3. Comparison of Bioanalysis Methods

The advantages of using electrochemical delectation for LC (LC-ECD) include the selectivity, low detection limit, and no need to derivatize the analyte before an analysis. The eluent used in this kind of detection should be electrically conductive (achieved by adding an auxiliary electrolyte). Using a buffer solution minimizes the influence of the sample matrix and acts as an electrolyte. This technique uses electrodes. High voltage causes rapid corrosion of their surface in complex biological matrices. This in turn leads to a decrease in sensitivity. Therefore, there is a need for sample purification to remove compounds leading to contamination of the electrode. Even 0.25 mL of sample is sufficient to analyze olanzapine concentration by LC-ECD. Linearity in the range of 0.2–150 ng/mL is possible. Unfortunately, the disadvantage is the long analysis time (4.2 min or longer).

The advantages of UV and DAD detection in LC are versatility, stability, no destructiveness of analytes, possibility of serial connection to other detectors (such as mass spectrometry), relative resistance to changes in flow/refractive index of mobile phases/temperature fluctuations (compatibility with gradient elution analysis), the many applications of UV detectors, and low maintenance requirements. The UV-VIS detector has one light-receiving section on the sample side, whereas the DAD has multiple photodiode arrays to acquire information even over a wide range of wavelengths in the same unit of analysis time. DAD enables simultaneous measurement of the entire UV spectrum at a specific sampling rate. The disadvantage of UV detectors is the limited application when organic solvents that absorb UV light are used for separation (the solvent must be compatible with UV detection). LC-DAD and LC-UV allow us to obtain linearity for olanzapine over a wide range (0–5000 ng/mL). Only 0.020 mL of sample is required for analysis. With this method, a shorter retention time (2.34 min) for olanzapine can be obtained than with the LC-ECD method.

LC-MS and LC-MS/MS enable the detection and identification of individual components of complex mixtures due to very high sensitivity and selectivity. ESI is the least destructive ionization method currently available in mass spectrometry. The advantages of LC-MS and LC-MS/MS include the possibility of simultaneous determination of several substances, the possibility of determining polar/high-molecular-weight compounds, a wide linear range of determinations, the previously mentioned very low detection limits, a very small sample needed for analysis (very small amount of injection directly onto the apparatus), and the ability to detect more compounds than when using gas chromatography (wide application). The disadvantages are the need for solubility of the analytes, use of standards and reagents of very high purity, and high cost of maintaining the equipment. Of all the studies listed in the tables, the smallest amount of biological sample was used (0.015 mL) and the shortest retention time (0.65 min) was obtained in the case of LC-MS/MS.

The last method described in this work is gas chromatography. Its advantages are fast analysis, small sample (<1 mL), high efficiency leading to high resolution, use of sensitive detectors (ppb), high accuracy (<1% RSD), non-destructive in the case of an MS detector, and high availability of spectral libraries for GC-MS. The disadvantages of this technique include, first of all, limitation to volatile samples or the need for derivatization, destructiveness of detectors (apart from the MS detector)/lack of application in analysis of samples, which are degraded in elevated temperatures (thermally unstable), the need for derivatization, not applicable in chromatography preparative, possibility of structural analysis only using an MS detector, limited capacity of peaks in the analysis of very complex samples, and over-sensitivity and -specificity compared LC-MS/MS. Moreover, direct analysis of a mixture of compounds in GC is difficult due to the interactions between the determined compounds and interactions between substances and the stationary phase of the chromatographic column.

## 5. Stability

**Figure 14:**
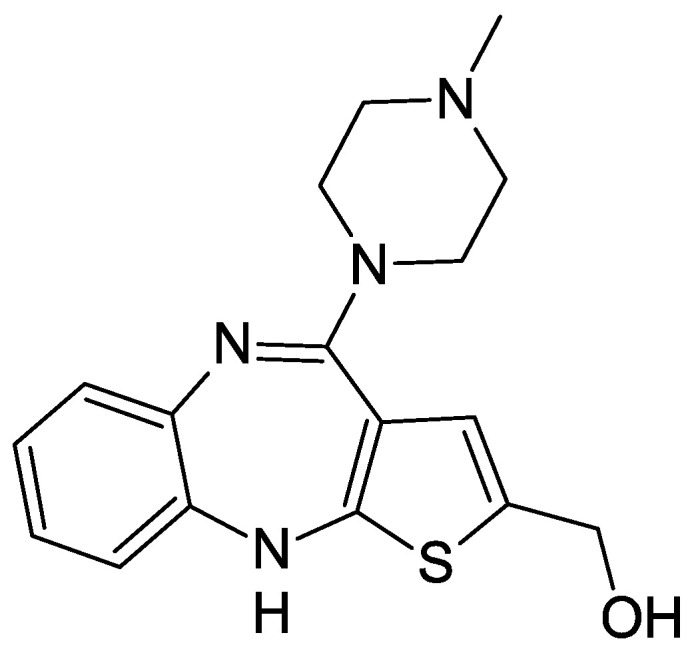
Olanzapine degradation product in water samples proposed by the team of E. Saar [137].

One of the key problems of olanzapine determination methods in biological material is its stability. Stability may be affected by storage conditions such as temperature, exposure to light, type of surface of the storage container, pH of the sample, enzymes present in it, anticoagulants, number of freeze–thaw cycles, and loss of solvent (due to evaporation or lyophilization effect). Moreover, instability can occur during any of the numerous steps of sample preparation [132,133]. When developing assay methods, these factors should be identified and managed to ensure that sample degradation does not jeopardize assay performance or underestimate drug concentrations [134]. Therefore, short-term and long-term stability studies should be performed for the standard as well as for real samples. Unfortunately, a large portion of the results of olanzapine stability studies are inconsistent and puzzling, and the exact conditions for conducting the stability test are not clearly defined [29]. For example, it has been reported that olanzapine can be stabilized by in vitro addition of stabilizing agents with antioxidant properties such as ascorbic acid [36,45,47,51,56,65,69,74,79,102,106], dithiothreitol (DTT), or tris(2-carboxyethyl)phosphine (TCEP) [133]. However, another group of authors described the addition of an antioxidant as unnecessary [43,44,62]. The team of H. Nozawa (2023) pointed out that the addition of ascorbic acid during sample collection carries the risk of reducing the concentration of olanzapine N-oxide as a result of its conversion to the parent compound, i.e., olanzapine itself. Moreover, N-oxides are relatively easily reduced, not only by compounds such as ascorbate but also under alkaline conditions during storage or extraction, and olanzapine N-oxide may decompose to the parent compound [114]. In some of the published studies, olanzapine was unstable in biological material, and the addition of ascorbic acid prevented the concentration from decreasing during storage. In contrast, other studies have shown the stability of olanzapine in samples, without the addition of an antioxidant. Conversely, other authors did not mention oxidation problems during sample preparation, but the mean inter-individual variability in serum olanzapine concentration in seven patients on stable doses was approximately 40% [135]. The teams of D. S. Fisher (2013) and T. N. Andreassen (2015) [29,136] responded to the need to conduct comprehensive studies on the impact of individual factors on the stability of olanzapine in various biological materials. The team of E. Saar (2012) identified one of the breakdown products of olanzapine in water samples, which is presented in Figure 14 [137]. This Figure shows the structure of olanzapine after protonation of the [M+H]+ precursor in the water environment; therefore, olanzapine has an additional OH group in its structure. Table 4 shows the summary of sixty-four olanzapine stability studies. The stability was checked in the following samples: human plasma, serum, human serum (with 0.25% ascorbic acid), saliva, human plasma (with 2.5% ascorbic acid), whole human blood, whole human blood (with 0.5% ascorbic acid), human blood (K3-EDTA anticoagulant), human blood (anticoagulant lithium heparin), whole blood (sodium ascorbate, 3 mM), human hemolyzed whole blood (with ascorbic acid, 333 mM), hemolyzed whole blood (ascorbic acid, 300 mg/L)/(DTT, 300 mg/L)/(TCEP, 300 mg/L), dried paper plasma spots, extracts of hair, nails, urine (sodium ascorbat, 3 mM), human milk, reconstituted samples, dry extract, Ringer’s fluid, propanol solutions, methanol solutions, solutions in 5 mM ammonium, formate–acetonitrile, rat plasma, rat brain (homogenized), and calf serum.

## 6. Conclusions

This review summarizes the analytical methods for the determination of olanzapine in biological material published in recent years. Efforts in the search for alternative biological matrices, enabling non-invasive collection of material for testing, were taken into account, and attention was paid to new trends in sample preparation and the evolution of analytical techniques. In recent years, many research teams have developed analytical methods that enable the quantitative determination of olanzapine concentration in biological material, not only during pharmacotherapy of schizophrenia but also in bioequivalence, pharmacokinetic, and toxicological studies. The ability to accurately analyze patients’ olanzapine concentrations in biological fluids in therapeutic drug monitoring provides healthcare professionals with valuable information on drug metabolism, which helps to improve therapeutic approaches and minimize drug side effects. Currently, the most commonly used technique in the bioanalysis of olanzapine is liquid chromatography combined with tandem mass spectrometry. It is thanks to the development of this sensitive and selective technique that olanzapine can be quantified not only in such conventional biological material as whole blood, plasma, and serum but also in alternative matrices such as hair or saliva. Although the first published studies focused mainly on the analysis of olanzapine alone, in the following years, with the development of bioanalytical methods, the focus was on creating multi-analyte analysis methodologies, including olanzapine metabolites and other antipsychotics. Gas chromatography, especially gas chromatography coupled with mass spectrometry (selective, repeatable, with high availability of spectral libraries), is a frequently used technique in bioanalysis, but it is rarely used for the determination of olanzapine and its metabolites. The main limitation is the volatility of olanzapine. Many of the sample preparation protocols cited are based on conventional techniques such as protein precipitation, LLE, or SPE. However, many authors have also used other modern sample preparation procedures such as SLE, MEPS, DPX, or fully automatic on-line SPE using column switching. The automated, miniaturized, and simplified sample preparation procedures combined with advanced instrumental techniques are the ideal approach for the analysis of olanzapine, its metabolites, and other antipsychotics, as this approach best provides valuable information about drug concentrations and potential biomarkers related to olanzapine activity and drug interactions in various biological matrices.

## Figures and Tables

**Table 1:** Olanzapine determination methods using LC-ECD.

Matrix	Sample Volume [mL]	Preparation	Mobile Phase	Elution, Flow	Analytes (Retention Time)	IS (Retention Time)	Linearity Range for Olanzapine [ng/mL], Correlation Coefficient for Olanzapine	Application, Reference, Matrix
human plasma	1.0	SPE	phosphate buffer (75 mM, pH 7)/MeOH/ACN (48:26:26)	isocratic 1.2 mL/min	olanzapine (7.0 min)	LY170222 (10.5 min)	0.25–50 >0.997	clinical research [74]
rat plasma	-	SPE	phosphate buffer (75 mM, pH 7)/MeOH/ACN (48:26:26)	isocratic 1.2 mL/min	2-hydroxymethylolanzapine (2.9 min) N-desmethyl olanzapine (3.9 min) olanzapine (6.6 min)	LY170222 (9.6 min)	1–100 >0.9912	pharmacokinetic studies [75]
human milk	1.0	SPE	phosphate buffer (75 mM, pH 7)/MeOH/ACN (48:26:26)	isocratic 1.2 mL/min	olanzapine (7.5 min)	LY170222 (11.5 min)	0.25–100 ≥0.9992	- [76]
rat brain tissue (homogenized)	0.5	LLE	phosphate buffer (75 mM, pH 7)/MeOH/ACN (48:26:26)	isocratic 1.2 mL/min	olanzapine (7.5 min)	LY170158 (10.5 min)	0.5–100 ≥0.9995	pharmacokinetic studies [46]
human plasma	0.5	SPE	phosphate buffer (15.4 mM, pH 3.8) + triethylamine (19.7 mM)/ACN (80:20)	isocratic 1.2 mL/min	N-desmethyl olanzapine (3.9 min) olanzapine (6.1 min)	LY170222 (12.6 min)	5–150 0.9996	therapeutic drug monitoring [62]
human plasma	0.25	SPE	phosphate buffer (8.9 mM) + triethylamine (7.18 mM)/MeOH/ACN (79.3:11:9.7)	isocratic 0.7 mL/min	N-desmethyl olanzapine (5.7 min) olanzapine (6.8 min)	LY170222 (15.6 min)	0.5–75 0.979	therapeutic drug monitoring [68]
human plasma	0.25	SPE	phosphate buffer (50 mM) + 0.175% triethylamine/MeOH (70:30)	isocratic 1.2 mL/min	N-desmethyl olanzapine (3.1 min) olanzapine (4.2 min)	LY170222 (6.9 min)	0.4–40 0.9993	therapeutic drug monitoring [105]
rat brain tissue (homogenized)	0.25	SPE	phosphate buffer (44 mM, pH 3.5) + 0.175% triethylamine/MeOH (79:21)	isocratic 1.2 mL/min	olanzapine (7.0 min)	LY170222 (13.8 min)	0.2–100 0.99993	pharmacokinetic studies [69]
human plasma	0.5	LLE	ammonium acetate (0.06 M, pH 5.9)/ACN/MeOH (40:41:37)	isocratic 0.69 mL/min	olanzapine (4.9 min)	clozapine (8.2 min)	0.313–25.00 =0.997	bioequivalence studies [47]
human plasma	0.5	SPE	phosphate buffer (50 mM, pH 5.7)/ACN/MeOH (67:22:11)	isocratic 1 mL/min	N-desmethyl olanzapine (5.88 min) olanzapine (8.04 min)	clozapine (26.27 min)	1–100 >0.999	therapeutic drug monitoring [63]

**Table 2:** Olanzapine determination methods using LC-UV and LC-DAD.

Matrix	Sample Volume [mL]	Preparation	Mobile Phase	Elution, Flow	Analytes (Retention Time)	IS (Retention Time)	Linearity Range for Olanzapine [ng/mL], Correlation Coefficient for Olanzapine	Application, Reference
human plasma	1.0	LLE	ammonium acetate (50 mM, pH 9.9)/MeOH (15:85)	isocratic 1.1 mL/min	olanzapine (3.58 min)	trifluoperazine (4.20 min)	0–469 1.0000	therapeutic drug monitoring [36]
human serum	1.2	on-line SPE	ammonium acetate (50 mM, pH 9.9)/MeOH (15:85)	isocratic 1.1 mL/min	olanzapine (3.58 min)	trifluoperazine (4.20 min)	0–1200 nmol/L 1.0000	therapeutic drug monitoring [102]
human plasma human urine	1.0	LLE	phosphate buffer (50 mM, pH 6.0)/ACN/MeOH (65:25:10)	isocratic 1.0 mL/min	olanzapine (approx. 9 min)	LY170222 (approx. 11 min)	1–5000 >0.98	bioequivalence and pharmacokinetic studies [42]
human plasma	0.1	on-line SPE (column switching)	H_2_O/ACN/tetramethylethylenediamine (62.6:37:0.4)	isocratic 1.5 mL/min	N-demethyl olanzapine (3.68 min) olanzapine (4.46 min)	fluperlapine (11.18 min)	10–180 >0.993	therapeutic drug monitoring [90]
human plasma	1.0	LLE	H_2_O/ACN/H_3_PO_4_/TEA (85.7:14:0.25:0.05)	isocratic 1.0 mL/min	olanzapine (5.4 min)	N- desmethyl clozapine (11.2 min)	2–150 µg/L ≥0.999	therapeutic drug monitoring [44]
human plasma	0.5	LLE	A: phosphate buffer (50 mM, pH 3.8) B: ACN	gradient 1.5 mL/min	olanzapine (4.0 min)	methylrisperidone (7.4 min)	10–1000 >0.997	clinical research [38]
human plasma	1.0	LLE	H_2_O:ACN (55:45) + heptanesulfonic acid sodium salt (0.009 M) and KH_2_PO_4_ (0.06 M, pH 2.7)	isocratic 1.0 mL/min	olanzapine (3.6 min)	clozapine (5.7 min)	5–100 1.0000	therapeutic drug monitoring [37]
human serum	0.099	on-line SPE (column switching)	H_2_O/ACN/tetramethylethylenediamine (62.1:37.5:0.4)	isocratic 1.3 mL/min	olanzapine (9.16 min)	fluperlapine (15.57 min)	10–170 ≥0.9807	therapeutic drug monitoring [91]
rat plasma	1.0	LLE	A: ammonium acetate (30 mM) + 0.05% TEA (pH 5.86) B: ACN	gradient 1.0 mL/min	olanzapine (5.90 min)	imipramine (12.05 min)	2–500 >0.9988	therapeutic drug monitoring [48]
human serum	0.25	on-line SPE (column switching)	A: potassium dihydrogen phosphate (pH 3.1)/MeOH (90:10) B: ACN	gradient	N-desmethyl olanzapine (10.22 min) olanzapine (10.89 min)		2.5–320 1.000 0	therapeutic drug monitoring [92]
rat brain tissue (microdialysates)	0.020	MEPS	A: ammonium acetate (10 mM) + 0.05% TEA (pH 3.00) B: ACN	gradient 4 μL/min	olanzapine (approx. 14.5 min) olanzapine N-oxide (approx. 17.5 min)	-	0.5–250 nmol/L >0.9950	therapeutic drug monitoring [83]
human plasma	-	protein precipitation	A: phosphate buffer (20 mM, pH 3.14) B: ACN	gradient 0.8 bmL/min	olanzapine (2.34 min)	carbamazepine (approx. 6.30 min)	0.125–50.0 µg/mL 0.9999	- [106]
human saliva	1.0	SPE	A: H_2_O + FA (pH 3.5) + 0.1% TEA B: ACN	gradient 0.6 mL/min	N-demethyl olanzapine (about 4.5 min) olanzapine (approx. 7 min)	chlordiazepoxide (approx. 18 min)	10–1000 0.9948	therapeutic drug monitoring [77]
human plasma	0.09	SPE	A: potassium dihydrogen phosphate (50 mM) B: ACN	gradient 1 mL/min	olanzapine (approx. 8.5 min)	-	1–50 0.9961	- [107]

**Table 3:** Olanzapine determination methods using LC-MS and LC-MS/MS.

Matrix	Sample Volume [mL]	Preparation	Mobile Phase	Elution, Flow	Source Type (Ionization) Mass Spectrometer Scan Types	Analytes (Retention Time)	IS (Retention Time)	Linearity Range for Olanzapine [ng/mL], Correlation Coefficient for Olanzapine	Application, Reference
human plasma human serum	0.5	SPE	A: ammonium acetate (100 mM) B: MeOH/iPOH (80:20)	gradient 1 mL/min	APCI(+)–MS/MS Multiple Reaction Monitoring (MRM) (*m*/*z*) precursor ion → (*m*/*z*) product ion 313.4 → 256.2 (OLA) 327.3 → 270.1 (IS)	olanzapine (approx. 6.5 min)	LY170222 (approx. 7 min)	0.25–50 >0.9859	- [64]
human serum	1.0	SPE	ACN: ammonium formate (50 mM, pH 3.0) (25:75)	isocratic 0.3 mL/min	APCI(+)–MS Selected ion monitoring (SIM) (*m*/*z*) precursor ion [OLA+H]^+^ 313 [IS+H]^+^ 327	olanzapine (approx. 3.48 min)	LY170222 (approx. 6.10 min)	1–1000 mg/L 0.9997	therapeutic drug monitoring [65]
human blood	0.25	LLE	A: ammonium acetate (100 mM) B: MeOH/iPOH:H_2_O (60:16:4)	gradient 1 mL/min	APCI(+)–MS/MS Multiple Reaction Monitoring (MRM) (*m*/*z*) precursor ion → (*m*/*z*) product ion 313.4 → 256.2 (OLA) 327.3 → 270.1 (IS)	olanzapine (approx. 5.90 min)	LY170158 (approx. 6.30 min)	5–500 ≥0.9983	- [45]
human plasma	-	On-line SPE (column switching)	ACN:0.1% FA (20:80)	isocratic 0.5 mL/min	ESI(+)–MS/MS Selected Reaction Monitoring (SRM) (*m*/*z*) precursor ion → (*m*/*z*) product ion 313 → 256 (OLA) 296 → 251 (IS)	olanzapine (2.44 min) N-demethyl clozapine (2.61 min)	dibenzepin (3.31 min)	5–300 µg/L ≥0.991	- [93]
human plasma	0.05	LLE	ammonium acetate (10 mM) + FA (2.7 mM)/CAN (53:47)	isocratic 0.16 mL/min	ESI(+)–MS Selected ion monitoring (SIM) (*m*/*z*) precursor ion [OLZ+H]^+^ 313 [IS+H]^+^ 286	olanzapine (4.80 min)	diazepam (7.44 min)	1–50 0.9993	therapeutic drug monitoring can be used in pharmacokinetic studies [35]
human serum with different anticoagulants	0.2	SPE	A: ACN/acetate ammonium (20 mM) (52:48) B: FA/ACN (0.1:100)	gradient 0.4 mL/min	ESI(+)–MS/MS Multiple Reaction Monitoring (MRM) (*m*/*z*) precursor ion → (*m*/*z*) product ion 312.9 → 256.0 (OLA) 316.1 → 256.0 (IS OLA)	N- desmethyl olanzapine (1.35 min) olanzapine (1.50 min)	N- desmethyl olanzapine-D8 (1.35 min) olanzapine-D3 (1.50 min)	0.1–50 0.9993	- [78]
human plasma	0.5	LLE	10 mM ammonium acetate + ACN (10:90)	isocratic 0.8 mL/min	ESI(+)–MS/MS Multiple Reaction Monitoring (MRM) (*m*/*z*) precursor ion → (*m*/*z*) product ion 313 → 256 (OLA) 383 → 337 (IS)	olanzapine (1.0 min)	loratadine (1.3 min)	0.1–30 0.998	studies of bioavailability and bioequivalence [34]
rat brain tissue (homogenized)	0.2	LLE	A: ammonium solution (5 mM, pH 6.1) B: ACN	gradient 0.3 mL/min	ESI (+)–MS/MS Multiple Reaction Monitoring (MRM) (*m*/*z*) precursor ion → (*m*/*z*) product ion 313→256 (OLA) 326→291 (IS)	olanzapine (6.70 min)	midazolam (7.96 min)	0.208–416.0 ng/g >0.997	- [48]
human blood	0.2 g	protein precipitation	A: ammonium hydroxide (5 mM, pH 9.0)/ACN (95:5) B: ACN	gradient 0.2 mL/min	ESI (+)–MS/MS Multiple Reaction Monitoring (MRM) (*m*/*z*) precursor ion → (*m*/*z*) product ion 313 → 256 (OLA) 296 → 251 (IS)	olanzapine (8.75) min	dibenzepin (8.77 min)	0–0.5 mg/kg >0.99	- [108]
human plasma	0.5	SPE	A: ammonium acetate (20 mM, pH 8.1) B: ACN	gradient 0.3 mL/min	ESI(+)–MS Selected ion monitoring (SIM) (*m*/*z*) precursor ion [OLZ+H]+ 313 [IS+H]+ 372	olanzapine (8.1 min)	remoxipride (6.2 min)	2–200	therapeutic drug monitoring [79]
human serum human cerebrospinal fluid	0.2	LLE	A: ammonium formate (10 mM) + 0.05% FA B: 0.05% FA in MeOH	gradient 0.3 mL/min	ESI(+)–MS/MS Multiple Reaction Monitoring (MRM) (*m*/*z*) precursor ion → (*m*/*z*) product ion 313 → 256 (OLA) 316 → 256 (IS)	N-demethyl olanzapine (1.9 min) olanzapine (2.1 min)	olanzapine-D3 (2.1 min)	5–100 serum 0.2–30 cerebrospinal fluid >0.999	therapeutic drug monitoring [49]
human plasma	0.5	SPE	ACN/H_2_O + 2% FA (70:30)	isocratic 0.5 mL/min	ESI(+)–MS/MS Multiple Reaction Monitoring (MRM) (*m*/*z*) precursor ion → (*m*/*z*) product ion 313.15 → 256.14 (OLA) 298.1 → 153.97 (IS)	olanzapine (2.17 min)	duloxetine (1.80 min)	0.12–25.03 0.9928	therapeutic drug monitoring [66]
human plasma	0.2	SPE	ACN/0.01% ammonia in ammonium formate solution (2 mM, pH 6.6) (85:15)	isocratic 0.9 mL/min	ESI(+)–MS/MS Multiple Reaction Monitoring (MRM) (*m*/*z*) precursor ion → (*m*/*z*) product ion 313.2 → 256.2 (OLA) 384.2 → 253.2 (IS)	olanzapine (2.36 min)	quetiapine (2.06 min)	0.1–40 ≥0.9996	bioequivalence studies [73]
rat brain tissue (homogenized)	0.02	on-line SPE (column-switching)	MeOH/ACN/H_2_O + 0.1% ammonium acetate (43:43:14)	isocratic 0.3 mL/min	ESI(+)–MS/MS Multiple Reaction Monitoring (MRM) (*m*/*z*) precursor ion → (*m*/*z*) product ion 313 →256 (OLA) 385 → 253 (IS)	olanzapine (approx. 4.0 min)	quetiapine (approx. 4.1 min)	0.085–17.34 0.9983	pharmacokinetic studies [94]
human plasma	0.5	LLE	ammonium acetate (10 mM, pH 4.0)/ACN (44:56)	isocratic 0.2 mL/min	ESI(+)–MS/MS Selected ion monitoring (SIM) (*m*/*z*) precursor ion [OLZ+H]^+^ 313.15 [IS+H]^+^ 383.00	olanzapine (4.2 min)	loratadine (3.1 min)	0.5–50 0.9987	bioequivalence studies [43]
human brain tissue	100 mg	precipitation +SPE	A: ammonium formate (pH 8.2) B: ACN	gradient 0.4 mL/min (0–9 min) 0.5 mL/min (9–25 min) 0.4 mL/min (25–30 min)	ESI (+)–MS/MS Multiple Reaction Monitoring (MRM) (*m*/*z*) precursor ion → (*m*/*z*) product ion 313 → 256 (OLA) 380 → 169 (IS)	olanzapine (8.8 min)	haloperidol-D4 (10.7 min)	2–8000 ng/g 0.996	toxicological studies [89]
human plasma	0.2	SPE	MeOH/acetate ammonium (2 mM) (90:10)	isocratic 1.0 mL/min	ESI(+)–MS/MS Multiple Reaction Monitoring (MRM) (*m*/*z*) precursor ion → (*m*/*z*) product ion 313 → 256 (OLA) 316 → 256 (ISOLA)	olanzapine (1.05 min)	oanzapine-D3 (1.05 min)	0.1–20 0.9991	pharmacokinetic studies [70]
human plasma	0.25	LLE	ACN/H_2_O + 30 mM ammonium formate (pH 5.0, 90:10)	isocratic 0.400 mL/min	ESI(+)–MS/MS Multiple Reaction Monitoring (MRM) (*m*/*z*) precursor ion → (*m*/*z*) product ion 313.19 → 256.12 (OLA) 298.14 → 154.09 (IS)	olanzapine (1.69 min)	duloxetine (1.72 min)	0.10–50 ≥0.99	pharmacokinetic studies [39]
human plasma	0.25	protein precipitation	A: 10 mM ammonium formate in H_2_O (pH 3.0) B: ACN	gradient 0.4 mL/min	ESI(+)–MS/MS Multiple Reaction Monitoring (MRM) (*m*/*z*) precursor ion → (*m*/*z*) product ion 313.2 → 256.2 (OLA) 321.2 → 261.2 (ISOLA)	olanzapine (0.63 min)	olanzapine-D3 (0.62 min)	0.5–400 0.997	therapeutic drug monitoring [26]
human plasma, serum, oral fluid, and hemolyzed whole blood	0.2	LLE	acetate ammonium in MeOH (50 mM, pH 6.0)	isocratic 0.5 mL/min	APCI(+)–MS/MS Selected Reaction Monitoring (SRM) (*m*/*z*) precursor ion → (*m*/*z*) product ion 313.2 → 256.2 (OLA) 321.2 → 261.2 (IS OLA)	olanzapine (5.17 min)	LY170222 (4.42 min)	2–200 >0.99	therapeutic drug monitoring [50]
human serum	0.2	LLE	A: ammonium acetate (10 mM, pH 3.7) B: ACN	gradient 0.5 mL/min	ESI(+)–MS/MS Multiple Reaction Monitoring (MRM) (*m*/*z*) precursor ion → (*m*/*z*) product ion 313.2 → 256.0 (OLA) 316.2 → 256.0 (ISOLA)	N-demethyl olanzapine (0.79 min) olanzapine (0.91 min)	N-demethyl olanzapine-D8 (0.78 min) olanzapine-D3 (0.90 min)	1–300 >0.99	therapeutic drug monitoring [51]
human plasma	0.4	LLE	A: 0.1% FA in H_2_O B: 0.1% FA in ACN (50:50)	isocratic 1.2 mL/min	ESI(+)–MS/MS Multiple Reaction Monitoring (MRM) (*m*/*z*) precursor ion → (*m*/*z*) product ion 313.1 → 256.1 (OLA) 278.1 → 260.2 (IS)	olanzapine (0.78 min)	venlafaxine (1.04 min)	1–20 ng/mL 0.9976	pharmacokinetic studies [52]
human plasma	0.05	protein precipitation	A: ammonium acetate (10 mM) + 0.05% FA (pH 3.5) B: MeOH + 0.05% FA	gradient 0.3 mL/min	ESI(+)–MS/MS Selected Reaction Monitoring (SRM) (*m*/*z*) precursor ion → (*m*/*z*) product ion 313.3 → 256.1 (OLA) 230.0 → 213.1 (IS)	olanzapine (3.11 min) N-desmethyl olanzapine (3.00 min)	4-amino-2-methyl-10H -thieno[2,3-b][1,5]-benzodiazepine (3.28 min)	0.2–120 0.9996	therapeutic drug monitoring [27]
human serum	0.1	protein precipitation	A: 0.1% FA in H_2_O B: MeOH	gradient 0.5 mL/min	ESI(+)–MS/MS Multiple Reaction Monitoring (MRM) (*m*/*z*) precursor ion → (*m*/*z*) product ion 313.1 → 256.1 (OLA) 316.1 → 256.1 (IS)	olanzapine (0.86 min)	olanzapine-D3 (0.86 min)	5–500 nM ≥0.9990	therapeutic drug monitoring [29]
human plasma	0.2	on-line SPE (column-switching)	A: ACN+ ammonium formate (10 mM) C: H_2_O + 0.01% FA	gradient 1.0 mL/min	ESI (+)–MS/MS Multiple Reaction Monitoring (MRM) (*m*/*z*) precursor ion → (*m*/*z*) product ion 313.0 → 256.2 (OLA) 256.2 → 167.2 (IS)	olanzapine (5.99 min)	diphenhydramine (7.35 min)	0.25–50 0.9998	therapeutic drug monitoring [95]
human oral fluid	0.5	LLE	A: ammonium acetate (10 mM, pH 3.7) B: ACN	gradient 0.5 mL/min	ESI(+)–MS/MS Multiple Reaction Monitoring (MRM) (*m*/*z*) precursor ion → (*m*/*z*) product ion 313.2 → 256.0 (OLA) 316.2 → 256.0 (ISOLA)	N-demethyl olanzapine (0.8 min) olanzapine (0.9 min)	N-demethyl olanzapine-D8 (0.8 min) olanzapine-D3 (0.9 min)	1.6–480 ≥0.995	therapeutic drug monitoring [19]
human plasma human blood	0.025 (human blood) 0.015 (human plasma)	SLE	A: ACN/H_2_O/TFA/acetic acid (85:15:0.025:0.5) B: ACN/H2O/TFA/acetic acid (95:5:0.025:0.5) (25:75)	isocratic 0.5 mL/min	ESI(+)–MS/MS Selected Reaction Monitoring (SRM) (*m*/*z*) precursor ion → (*m*/*z*) product ion 313.2 → 256.1 (OLA) 317.2 → 212.7 (ISOLA)	olanzapine (3.37 min)	olanzapine-D4 (3.36 min)	0.100–100 quantified in human plasma only 0.9994	therapeutic drug monitoring [56]
human plasma	1.0	SPE	H_2_O + 0.1% FA: ACN + 0.1% FA (70:30)	isocratic 0.4 mL/min	ESI(+)–MS/MS Multiple Reaction Monitoring (MRM) (*m*/*z*) precursor ion → (*m*/*z*) product ion 313 → 256 (OLA) 411 → 191 (IS)	olanzapine (1.21 min)	risperidone (0.78 min)	5–50 0.9971	toxicological studies [67]
human plasma	0.2	LLE	MeOH/ammonium formate (20 mM) (82.5:17.5)	isocratic 1.0 mL/min	ESI(+)–MS/MS Multiple Reaction Monitoring (MRM) (*m*/*z*) precursor ion → (*m*/*z*) product ion 313.10 → 256.05 (OLA) 316.05 → 256.05 (ISOLA)	olanzapine (2.68 min)	olanzapine-D3 (2.66 min)	0.2–25 >0.9900	pharmacokinetic studies [32]
human plasma	0.45	LLE	A: ACN B: 0.2% FA in H_2_O	gradient 0.6 mL/min	ESI(+)–MS/MS Multiple Reaction Monitoring (MRM) (*m*/*z*) precursor ion → (*m*/*z*) product ion 313.0 → 256.0 (OLA) 294.2 → 225.1 (IS)	olanzapine (2.65 min)	anastrozole (7.45 min)	0.5–100 0.998	therapeutic drug monitoring [33]
human plasma	0.5	SPE	A: ammonium formate (2 mM, pH 2.7) B: ACN	gradient 0.8 mL/min	ESI (+) Multiple Reaction Monitoring (MRM) (*m*/*z*) precursor ion → (*m*/*z*) product ion 313.2 → 198.2 (OLZ) 453.3 → 230.2 (IS)	olanzapine (2.34 min)	repaglinide	5–150 0.99915	therapeutic drug monitoring [71]
human plasma	0.2	protein precipitation	A: ammonium formate (2 mM) + 0.2% FA B: ACN + ammonium formate (2 mM) + 0.2% FA	gradient 0.4 mL/min	WHEN (+)/WHEN (−)–MS/MS Multiple Reaction Monitoring (MRM). (*m*/*z*) precursor ion → (*m*/*z*) product ion 313.1 → 256.2 (OLA) 298.3 → 103.0 (IS OLA)	olanzapine (3.50 min)	topiramate-D12 (6.0 min) trimipramine-D3 (6.51 min)	0.01–0.5 0.9997	- [109]
rat plasma	0.1	SLE	ACN/ammonium formate (15 mM) + 0.05% TFA (60:40)	isocratic 1 mL/min	ESI(+)–MS Selected ion monitoring (SIM) (*m*/*z*) precursor ion [OLZ+H]+ 313 [IS+H]+ 441	olanzapine (5.82 min)	risperidone (3.10 min)	2–5000 ng/mL 0.9989	therapeutic drug monitoring [57]
human plasma	0.095	protein precipitation	A: MeOH B: 0.005% heptafluorobutyric acid + ammonium formate (10 mM)	gradient 0.5 mL/min	ESI(+)–MS/MS Multiple Reaction Monitoring (MRM) (*m*/*z*) precursor ion → (*m*/*z*) product ion 313.2 → 256.1 (OLA) 316.2 → 256.1 (IS OLA)	olanzapine (1.97 min)	olanzapine-D3 (1.97 min)	0.1–20 0.9989	bioequivalence studies [110]
human plasma	0.5	LLE	H_2_O + 0.1% trifluoroacetic acid/ACN (20:80)	isocratic 1 mL/min	ESI(+)–MS Selected ion monitoring (SIM) (*m*/*z*) precursor ion [OLZ+H]+ 313.1 [IS+H]+ 429.4	olanzapine (1.53 min)	irbesartan (2.12 min)	2–300 >0.9993	therapeutic drug monitoring [53]
human plasma	0.2	µSPE	A: 0.2% formic acid (pH 3.0) D: 0.2% formic acid/ACN (65:35)	gradient 0.6 mL/min	APCI(+)–MS/MS Selected Reaction Monitoring (SRM) (*m*/*z*) precursor ion → (*m*/*z*) product ion 313.2 → 256.2 (OLA) 317.3 → 256.2 (IS OLA)	olanzapine (1.941 min)	olanzapine-13C-D3 (1.940 min)	0.70–60 ng/mL >0.99	therapeutic drug monitoring [72]
human plasma	0.09	LLE	formate ammonium (5 M, pH of 4.7) and 85.5% of acetonitrile	isocratic 1.2 mL/min	ESI(+)–MS/MS Multiple Reaction Monitoring (MRM) (*m*/*z*) precursor ion → (*m*/*z*) product ion 313.1 → 256.1 (OLA) 278.2 → 260.2 (IS)	olanzapine (4.0 min)	venlafaxine (about 2.9 min)	1.0–20	bioequivalence and pharmacokinetic studies [40]
human saliva	0.5	SPE	A: ammonium formate (2 mM) B: ACN	gradient 0.25 mL/min	ESI(+)–MS/MS Multiple Reaction Monitoring (MRM) (*m*/*z*) precursor ion → (*m*/*z*) product ion 313.5 → 256.3 (OLA) 321.5 → 261.3 (IS)	olanzapine (2.41 min)	olanzapine-D8 (2.41 min)	1–500 >0.9980	therapeutic drug monitoring [80]
human serum	0.050	protein precipitation	A: ammonium formate (2 mM) B: MeOH	gradient 0.5 mL/min	ESI(+)–MS/MS Multiple Reaction Monitoring (MRM) (*m*/*z*) precursor ion → (*m*/*z*) product ion 313.2 → 256.1 (OLA) 321.2 → 261.2 (ISOLA)	olanzapine (1.30 min)	olanzapine-D8 (1.26 min)	5–500 ≥0.9946	therapeutic drug monitoring [25]
protein and melanin hair fraction	10 mg	SPE	A: 0.1% FA in H_2_O B: ACN	gradient 0.8 mL/min	ESI(+)–MS/MS Multiple Reaction Monitoring (MRM) (*m*/*z*) precursor ion → (*m*/*z*) product ion 313.2→256.1 (OLA) 321.2→260.1 (IS OLA)	desmethyl olanzapine (0.82 min) olanzapine (0.83 min)	N-desmetyl olanzapine-D8 (0.73 min) olanzapine-D8 (0.83 min)	40–1600	therapeutic drug monitoring [81]
human hair	10 mg	LSE	A: ammonium formate (5 mM, pH 3.0) B: 0.1% FA in ACN	gradient 0.4 mL/min	ESI (+)–MS/MS Multiple Reaction Monitoring (MRM) (*m*/*z*) precursor ion → (*m*/*z*) product ion 313 → 256 (OLA) 316 → 87 (IS)	olanzapine N-desmethyl olanzapine	olanzapine-D3	0.005–10.0	therapeutic drug monitoring [111,112]
dry plasma spots	100 µL plasma per disk	protein precipitation	A: 0.2% ammonium acetate (1M) + H_2_O/MeOH (95:5) B: 0.2% ammonium acetate (1M) + H_2_O/MeOH (5:95)	lower gradient 0.5 mL/min	ESI (+)–MS/MS Multiple Reaction Monitoring (MRM). (*m*/*z*) precursor ion → (*m*/*z*) product ion 313.1→256.1 (OLA) 316.1→256.1 (IS OLA)	olanzapine (1.04 min)	olanzapine-D3 (1.04 min)	7.3–151.0 >0.980	therapeutic drug monitoring [14]
nails hair	30 mg of nails 25 mg hair	SPE	A: ammonium formate + 0.1% FA B: ACN	gradient 0.3 mL/min	ESI(+)–MS/MS Multiple Reaction Monitoring (MRM) (*m*/*z*) precursor ion → (*m*/*z*) product ion 313.23→256.2 (OLA) 321.37→261.3 (ISOLA)	olanzapine (1.2 min)	olanzapine-D8 (1.3 min)	10–10,000 pg/mg ≥0.99	therapeutic drug monitoring [82]
human plasma	0.1	SLE	A: H_2_O + 0.1% FA B: MeOH	gradient 0.3 mL/min	ESI(+)–MS/MS Multiple Reaction Monitoring (MRM) (*m*/*z*) precursor ion → (*m*/*z*) product ion 313.2→256.1 (OLA) 415.3→195.2 (IS OLA)	olanzapine (3.322 min)	risperidone-D4 (6.929 min)	1–100 >0.995	therapeutic drug monitoring [58]
human plasma	0.2	LLE	A: H_2_O + 0.1% FA B: ACN/H_2_O + 0.1% FA (9:1)	gradient 0.5 mL/min.	ESI(+)–MS/MS Multiple Reaction Monitoring (MRM) (*m*/*z*) precursor ion → (*m*/*z*) product ion 313.2→256.0 (OLA) 316.2→256.0 (ISOLA)	desmethyl olanzapine (1.88 min) olanzapine (1.93 min)	desmethyl olanzapine-D8 (1.91 min) olanzapine-D3 (1.95 min)	1.0–512.0	therapeutic drug monitoring [41]
human serum	1.0	Pipette-tip micro-SPE	A: 0.1% FA + ammonium acetate (10 mM) B: ACN	gradient 0.3 mL/min	ESI (+)–MS Selected ion monitoring (SIM) (*m*/*z*) precursor ion [OLZ+H]^+^ 313.1476	olanzapine (2.9 min)	-	0.5–400 0.996	therapeutic drug monitoring [113]
postmortem human blood	0.1	SLE	A: 0.1% FA + ammonium formate (10 mM) B:0.1% FA + ammonium formate (10 mM) in MeOH	gradient 0.5 mL/min	ESI (+)/ESI (−)–MS/MS Selected Reaction Monitoring (SRM). (*m*/*z*) precursor ion → (*m*/*z*) product ion 313.2 → 84.3 (OLA) 290.2 → 198.2 (IS OLA)	olanzapine (2.36 min)	diazepam-D5 (4.52 min)	5–5000 0.998	toxicological studies [59]
human plasma	0.5	LLE	MeOH/0.5% FA (60:40)	isocratic 0.5 mL/min	ESI(+)–MS/MS Multiple Reaction Monitoring (MRM) (*m*/*z*) precursor ion → (*m*/*z*) product ion 313.2→256.1 (OLA) 327→270 (ISOLA)	olanzapine (0.9 min)	clozapine (1 min)	1–20 0.9976	bioequivalence studies [54]
human body fluids (blood, pericardial fluid, gastric contents, bile, and urine)	0.04	1. protein precipitation 2. LLE	A: ammonium acetate (10 mM) B: MeOH	gradient 0.2 mL/min	ESI(+)–MS/MS Multiple Reaction Monitoring (MRM) (*m*/*z*) precursor ion → (*m*/*z*) product ion 313→256 (OLA) 321.2→261.1 (IS)	olanzapine N- desmethyl olanzapine 2- OH-methylolanzapine olanzapine N- oxide	olanzapine-D8	0.05–10 >0.99	- [114]

**Table 4:** Summary of olanzapine stability studies.

Reference	Matrix	Conditions	Result
[75]	rat plasma	−70 °C (19 weeks)	stable
[64]	human plasma	room temperature (24 h) −70 °C (59 days) freeze–thaw (3 cycles) extracts (24 h)	stable
	human serum	room temperature (24 h) −70 °C (59 days) freeze–thaw (3 cycles) extracts (24 h)	
	propanol solutions	−20 °C (35 days)	
[76]	human milk	−70 °C (10 months) freeze–thaw (3 cycles) extracts (48 h)	stable
[65]	human serum	−20 °C (30 days) extracts: −20 °C (30 days)	stable
[36]	human serum (with 0.25% ascorbic acid)	room temperature (24 h) 4–8 °C (2 weeks)	unstable without ascorbic acid after 1 week at 4–8 °C and 24 h at ambient temperature
[68]	methanol solutions	−20 °C (2 months)	stable
[46]	rat brain (homogenized)	room temperature (2 h, 4 h) −70 °C (100 days) freeze–thaw (2 cycles) extracts (48 h)	unstable at room temperature after 4 h
[44]	human plasma	4 °C (7 days) −20 °C (3 months)	stable
[45]	human hemolyzed whole blood (with ascorbic acid, 333 mM)	room temperature (24 h) −70 °C (7 days) extracts (48 h)	stable
	propanol solutions	−20 °C (35 days) freeze–thaw (2 cycles)	
[38]	human plasma	room temperature (24 h) 4 °C (72 h) −20 °C (30 days) freeze–thaw (3 cycles) extracts: 4 °C (24 h)	unstable at ambient temperature in daylight after 24 h
[47]	human plasma	−70 °C (5 months) room temperature (4 h) freeze–thaw (3 cycles)	stable
	reconstituted sample	room temperature (18 h)	
[69]	methanol solutions	−80 °C (1 year)	stable
[105]	methanol solutions	−80 °C (1 year)	stable
[138]	human plasma	room temperature (14 days) −20 °C (12 months, repeated freeze–thaw)	unstable at room temperature after 7 days and at −20 °C with repeated freezing and thawing
[35]	human plasma (with 2.5% ascorbic acid)	room temperature (24 h) −20 °C (30 days) freeze–thaw (3 cycles)	unstable without ascorbic acid at room temperature after 24 h, and in repeated freeze–thaw cycles
	methanol solution	−20 °C (3 months)	
[91]	human plasma	room temperature (7 days) 20 °C (3 months)	unstable at ambient temperature after 7 days
[34]	human plasma	−50 °C (3 weeks)	stable
	methanol solutions	4–8 °C (4 months)	
[48]	solutions in 5 mM ammonium formate–acetonitrile	−20 °C (3 months)	unstable at room temperature after 12 h
	rat brains (homogenized)	room temperature (2 h, 12 h) freeze–thaw (3 cycles) 24 h extracts	
[108]	human whole blood (with 0.5% ascorbic acid)	−20 °C (12 months) extract: room temperature (3 days)	stable
[79]	human plasma	room temperature (24 h, 72 h) 4 °C (72 h) −20 °C (2 months) freeze and thaw (1 cycle, 3 cycles) extracts: room temperature (36 h) 4 °C (36 h)	unstable at room temperature and 4 °C after 72 h, in extracts at room temperature, at 4 °C after 36 h, and after 3 freeze–thaw cycles
[49]	human plasma	−20 °C (12 months) freeze–thaw (3 cycles)	unstable extracts after a freeze–thaw or storage cycle fridge 4 °C (24 h)
[139]	methanol solutions	room temperature 4–8 °C (1 year)	unstable after 1 month at ambient temperature and after 3 months at 4–8 °C
[73]	human plasma	room temperature (24 h) 20 °C (90 days) freeze–thaw (6 cycles)	stable
	methanol solutions	4 °C (41 days) extracts: room temperature (45 h) 4 °C (97 h)	
[137]	whole human blood	20 °C (10 weeks) 4 °C (10 weeks) −20 °C (10 weeks) −60 °C (10 weeks)	unstable in all conditions
[66]	human plasma	room temperature (6 h) freezing and thawing (5 cycles) 10 °C (24 h) −70 °C (45 days)	stable
	dry extract	2–8 °C (24 h)	
[94]	Ringer’s fluid	room temperature (12 h) −70 °C (45 days) freezing and thawing (3 cycles)	stable
[43]	human plasma	room temperature (8 h) −20 °C (15 days) 15 °C (24 h)	stable
	methanol solutions	room temperature (24 h) 4 °C (15 days)	
[89]	propanol solutions human brains (homogenized)	−70 °C (3 months) extracts (24 h) freezing and thawing (3 cycles)	unstable calibration solutions at −42 °C after 24 h
[70]	methanol solutions	room temperature (23 h) 2–8° (5 days)	stable
	human plasma	room temperature (7 h) −20 °C (21 days) −80 °C (6 days) freezing and thawing (5 cycles) extracts (58 h)	
[39]	human plasma	room temperature (5.22 h) −70 °C (22 days) freezing and thawing (4 cycles) extract (24 h) dry extract (24 h) wet extract (24 h)	stable
[26]	human blood (K3-EDTA anticoagulant)	room temperature (72 h) 4 °C (72 h)	stable
	human blood (anticoagulant lithium heparin) human plasma	room temperature (72 h) 4 °C (72 h) room temperature (72 h) 4 °C (72 h) −20 °C (3 months) freezing and thawing (3 cycles) extracts: 10 °C (72 h)	
[51]	human serum	room temperature (2 h, 3 h) −20 °C (3 months) freezing and thawing (4 cycles) extracts (every 24 h for 5 days)	stable
[52]	human plasma	room temperature (6 h) 4 °C (5 days) −70 °C (56 days) freezing and thawing (3 cycles) extracts (8 h)	stable
[27]	human plasma	room temperature (2 h) freezing and thawing (3 cycles) −70 °C (40 days) extracts: 4 °C (18 h)	stable
[29]	enriched human serum	room temperature, darkness and 3000 lux (0 h, 2 h, 4 h, 8 h, 24 h, 48 h) 4–8°, dark and 550 lux (0 h, 24 h, 48 h, 4 days, 7 days) −20 °C darkness (3 months, 6 months) freezing and thawing (3 cycles) −70 °C (40 days) extracts: 4–8 °C (24 h, 72 h)	unstable in spiked serum at room temperature, 3000 lux after 4 h, at 4–8 °C, 550 lux after 7 days, in patient serum at room temperature, 3000 lux after 48 h, and at 4–8 °C, 550 lux after 7 days
	whole human blood	room temperature, darkness and 3000 lux (0 h, 2 h, 4 h, 8 h, 24 h, 48 h)	
	serum collected from patients	room temperature, darkness and 3000 lux (0 h, 2 h, 4 h, 8 h, 24 h, 48 h) 4–8 °C, dark (0 h, 24 h, 48 h, 8 days) 4–8 °C, 550 lux (0 h, 72 h, 7 days, 14 days) −35 °C, dark (0 h, 24 h, 48 h, 72 h, 96 h)	
[95]	human plasma	room temperature (6 h) 4 °C (24 h) −80 °C (30 days) freezing and thawing (3 cycles)	stable
[13]	saliva	4 °C (2 days, 5 days, 7 days)	stable
[56]	human plasma	room temperature (6 h) on ice (31 h) −20 °C (614 days) −70 °C (614 days) freeze and thaw, −20 °C (5 cycles) freeze and thaw, −70 °C (5 cycles) extract 2–8 °C (267 h)	stable
	whole human blood	on ice (24 h) −20 °C (108 days) −70 °C (18 days) freeze and thaw, −20 °C (5 cycles) freeze and thaw, −70 °C (5 cycles) extract 2–8 °C (61 h)	
[67]	human plasma	−20 °C (12 months)	stable
[32]	human plasma	room temperature (24 h) −70 °C (21 days) freezing and thawing (3 cycles) extracts (48 h)	stable
[33]	human serum	freezing and thawing (3 cycles)	stable
[71]	human plasma	room temperature (6 h) −20 °C (1 month) freezing and thawing (4 cycles) extracts (24 h)	stable
[57]	rat plasma	room temperature (6 h) −20 °C (30 days) freeze and thaw, −20 °C to 25 °C (1 cycle) extracts (24 h)	stable
[110]	human plasma	room temperature (24 h) freeze and thaw, −80 °C (4 cycles) −80 °C (62 days) extracts (31 h)	stable
[53]	human plasma	room temperature (24 h, 48 h, 72 h) 4 °C (24 h, 48 h, 72 h) −20 °C (24 h, 48 h, 72 h) freezing and thawing (4 cycles)	unstable at room temperature after 48 h
[72]	human plasma	room temperature (24 h) 4 °C (24 h) −80 °C (1 month, 62 days) freeze and thaw, −80 °C (3 cycles) extracts 19 °C	stable
[80]	saliva	8 °C (72 h) –21 °C (72 h) extracts: 6 °C (72 h)	stable
[25]	human serum	room temperature (24 h) −20 °C (8 days) freezing and thawing (3 cycles) extracts: 4 °C (24 h)	stable
[58]	human serum	room temperature (24 h) 4 °C (24 h, 48 h) −20 °C (7 days)	sable
[81]	extract of hair	4–8 °C (3 days)	stable
[14]	dried papers plasma spots	room temperature (0 days, 3 days, 8 days)	stable
[82]	nails and hair extracts	6 °C (72 h)	stable
[41]	human plasma	room temperature (3 h) −20 °C (3 months) freezing and thawing (3 cycles) extracts (72 h)	stable
[113]	human serum	room temperature (12 h) 4 °C (7 days)	stable
[59]	whole human blood	room temperature (4 h) −80 °C (28 days) freezing and thawing (3 cycles)	stable
[114]	urine (sodium ascorbate, 3 mM)	room temperature (1 day, 2 days, 4 days, 7 days) 4 °C (1 day, 2 days, 4 days, 7 days) −30 °C (3 months) freezing and thawing (3 cycles) extracts: 4 °C (7 days) 10 °C (4 days)	
	whole blood (sodium ascorbate, 3 mM)	room temperature (1 day, 2 days, 4 days, 7 days) 4 °C (1 day, 2 days, 4 days, 7 days) −30 °C (3 months) freezing and thawing (3 cycles) extracts: 4 °C (7 days) 10 °C (4 days)	unstable with ascorbate addition at room temperature after 1 day
	urine	room temperature (1 day, 2 days, 4 days, 7 days) 4 °C (1 day, 2 days, 4 days, 7 days) −30 °C (3 months) freezing and thawing (3 cycles)	
	full blood	room temperature (1 day, 2 days, 4 days, 7 days) 4 °C (1 day, 2 days, 4 days, 7 days) −30 °C (3 months)	unstable in whole blood without ascorbate addition at room temperature
[83]	Ringer’s solution	5 °C (24 h) −80 °C (20 days) freezing and defrosting (3 cycles)	stable
[107]	human plasma	room temperature (24 h) −20 °C (2 weeks) freezing and thawing (3 cycles)	stable
[77]	saliva	8 °C (-) −21 °C (-) extracts: 15 °C (72 h)	stable
[107]	human plasma	4 °C (24 h) −20 °C (7 days) freeze and thaw, −20 °C to 25 °C (1 cycle)	stable
[129]	human plasma	−40 °C (0 days, 15 days, 30 days, 90 days)	stable
[128]	human plasma	room temperature, darkness (4 days) −18 °C (4 days)	unstable at room temperature after 7 days
[85]	human urine	room temperature (15 h, 48 h, 88 h) −24 °C (3 weeks) freezing and thawing (6 cycles)	unstable at room temperature after 48 h
[136]	methanolic stock solutions	2–8 °C (6 months) −20 °C (1 year)	not stable in methanol solution at 2–8 °C after 6 months
	human plasma	room temperature (5 days) 2–8 °C (1 month) −20 °C (1 year, 2 years) freezing and thawing (3 cycles)	not stable in human plasma at room temperature after 4 days, at 2–8 °C after 3 weeks, at −20 °C after 2 years
	hemolyzed whole blood	room temperature (5 days) 2–8 °C (1 month) −20 °C (1 year, 2 years) freezing and thawing (3 cycles)	not stable in hemolyzed whole blood at room temperature after 2 days, at 2–8 °C after 1 week, at −20 °C after 2 months, and after three freeze–thaw cycles
	hemolyzed whole blood (ascorbic acid, 300 mg/L)/(DTT, 300 mg/L)/(TCEP, 300 mg/L)	−20 °C (1 month) freezing and thawing (3 cycles)	the ascorbic acid-added solutions showed the lowest loss of olanzapine after 23 days at −20 °C and freeze–thaw compared to the DTT and TCEP solutions, which were similar to the unsupplemented solutions
	oral fluid	room temperature (2 days) 2–8 °C (1 week) −20 °C (2 months) freezing and thawing (3 cycles)	not stable in oral fluid at room temperature after 2 days, at 2–8 °C after 1 week, at −20 °C after 2 months, and after three freeze–thaw cycles
	human serum and calf serum	room temperature (2 days) 2–8 °C (1 week, 1 month) −20 °C (9 months)	not stable in human serum and calf serum at room temperature after 2 days unstable in human serum at 2–8 °C after 5 days and in calf serum after 3 weeks unstable in human serum at −20 °C after 9 months

## Data Availability

Data sharing is not applicable.
